# A New Mixed-Valence Mn(II)Mn(III) Compound With Catalase and Superoxide Dismutase Activities

**DOI:** 10.3389/fchem.2018.00491

**Published:** 2018-11-05

**Authors:** Rafael O. Costa, Sarah S. Ferreira, Crystiane A. Pereira, Jeffrey R. Harmer, Christopher J. Noble, Gerhard Schenk, Roberto W. A. Franco, Jackson A. L. C. Resende, Peter Comba, Asha E. Roberts, Christiane Fernandes, Adolfo Horn Jr.

**Affiliations:** ^1^Laboratório de Ciências Químicas, Universidade Estadual do Norte Fluminense Darcy Ribeiro, Campos dos Goytacazes, Brazil; ^2^Instituto Federal Fluminese, Campos dos Goytacazes, Brazil; ^3^Centre for Advanced Imaging, University of Queensland, Brisbane, QLD, Australia; ^4^School of Chemistry and Molecular Biosciences, University of Queensland, Brisbane, QLD, Australia; ^5^Laboratório de Ciências Físicas, Universidade Estadual do Norte Fluminense Darcy Ribeiro, Campos dos Goytacazes, Brazil; ^6^Instituto de Ciências Exatas e da Terra, Campus Universitário do Araguaia, Universidade Federal do Mato Grosso, Barra do Garças, Brazil; ^7^Anorganisch-Chemisches Institut, Universität Heidelberg, Heidelberg, Germany; ^8^Interdisziplinäres Zentrum für Wissenschaftliches Rechnen, Heidelberg, Germany

**Keywords:** catalase, superoxide dismutase, tripodal ligand, mix-valent manganese, polymeric manganese, reaction mechanism

## Abstract

The synthesis, X-ray molecular structure, physico-chemical characterization and dual antioxidant activity (catalase and superoxide dismutase) of a new polymeric mixed valence Mn(III)Mn(II) complex, containing the ligand H_2_BPClNOL (N-(2-hydroxybenzyl)-N-(2-pyridylmethyl)[(3-chloro)(2-hydroxy)] propylamine) is described. The monomeric unit is composed of a dinuclear Mn(II)Mn(III) moiety, [Mn(III)(μ-HBPClNOL)(μ-BPClNOL)Mn(II)(Cl)](ClO_4_)·2H_2_O, **1**, in which the Mn ions are connected by two different bridging groups provided by two molecules of the ligand H_2_BPClNOL, a phenoxide and an alkoxide group. In the solid state, this mixed valence dinuclear unit is connected to its neighbors through chloro bridges. Magnetic measurements indicated the presence of ferromagnetic [*J* = +0.076(13) cm^−1^] and antiferromagnetic [*J* = −5.224(13) cm^−1^] interactions. The compound promotes O${}_{2}^{•-}$ dismutation in aqueous solution (IC_50_ = 0.370 μmol dm^−3^, *k*_cat_ = 3.6x10^6^ M^−1^ s^−1^). EPR studies revealed that a high-valent Mn(III)-O-Mn(IV) species is involved in the superoxide dismutation catalytic cycle. Complex **1** shows catalase activity only in the presence of a base, e.g., piperazine or triethylamine. Kinetic studies were carried out in the presence of piperazine and employing two different methods, resulting in *k*_cat_ values of 0.58 ± 0.03 s^−1^ (detection of O_2_ production employing a Clark electrode) and 2.59 ± 0.12 s^−1^ (H_2_O_2_ consuption recorded via UV-Vis). EPR and ESI-(+)-MS studies indicate that piperazine induces the oxidation of **1**, resulting in the formation of the catalytically active Mn(III)-O-Mn(IV) species.

## Introduction

The best described and studied forms of reactive oxygen species (ROS) are the superoxide anion (O${}_{2}^{•-}$) and hydrogen peroxide (H_2_O_2_), which can produce the extremely reactive hydroxyl radical (HO^•^). Although performing key roles in biochemical processes such as the cell signaling, gene expression, and immune response, these oxidants also induce damage on cellular constituents, causing DNA, protein and lipid oxidation (Hancock et al., 2001; Halliwell, 2006; Morano et al., 2012). The uncontrolled generation of ROS has been related to many pathologies, including neurodegenerative disorders (Alzheimer's disease, amyotrophic lateral sclerosis, etc.) and is also thought to have an important action in the aging progression (Lane, 2003; McCord and Edeas, 2005; Valko et al., 2007).

Complex organisms such as human beings are able to co-exist with free radicals and have established pathways to employ such ROS as oxidation/reduction switches, in a process known as redox signaling (Allen and Tresini, 2000; Lane, 2003). Hence, a certain level of oxidation performed by free radicals is required in biosystems, but increased oxidative levels may result in damages to the normal functioning of biological systems, resulting in pathophysiological conditions.

As a protection stratagem to counter the deleterious properties of ROS, aerobic organisms have developed antioxidant metalloenzymes, e.g., glutathione peroxidase (GPx), catalases (CATs), and superoxide dismutases (SODs) (Costa and Moradas-Ferreira, 2001; Valko et al., 2007). Whereas GPx and CATs act on H_2_O_2_, SODs induce superoxide dismutation. GPx contains selenium in the active site (Lu and Holmgren, 2009) while CATs possess an iron(III) heme prostetic cofactor or a dinuclear manganese active site (Bravo et al., 1999; Antonyuk et al., 2000). In SODs, iron, manganese, copper/zinc or nickel have been reported at the active site (Tainer et al., 1982; Ludwig et al., 1991; Kerfeld et al., 2003; Barondeau et al., 2004). SOD is assumed to be the main mediator to control the damaging effects of the superoxide anion *in vivo*. However, several practical restrictions (large size, low cell permeability, short circulating half-life, antigenicity, high manufacturing costs) have restricted the usage of SODs as a possible clinical treatment (McCord and Edeas, 2005).

An alternative to the use of antioxidant metalloenzymes to decrease the level of ROS is the development of synthetic compounds which may mimic the activity of such enzymes (Mahammed and Gross, 2011). Several biomimetics that can decompose ROS produced during oxidative stress (e.g., using metal ion ligands such as salen, porphyrins, corroles, or non-aromatic macrocycles) have already been reported (Doctrow et al., 2002; Day, 2007; Eckshtain et al., 2009; Batinić-Haberle et al., 2010; Kupershmidt et al., 2010; Tovmasyan et al., 2015; Weekley et al., 2017; Signorella et al., 2018).

**Figure 1:**
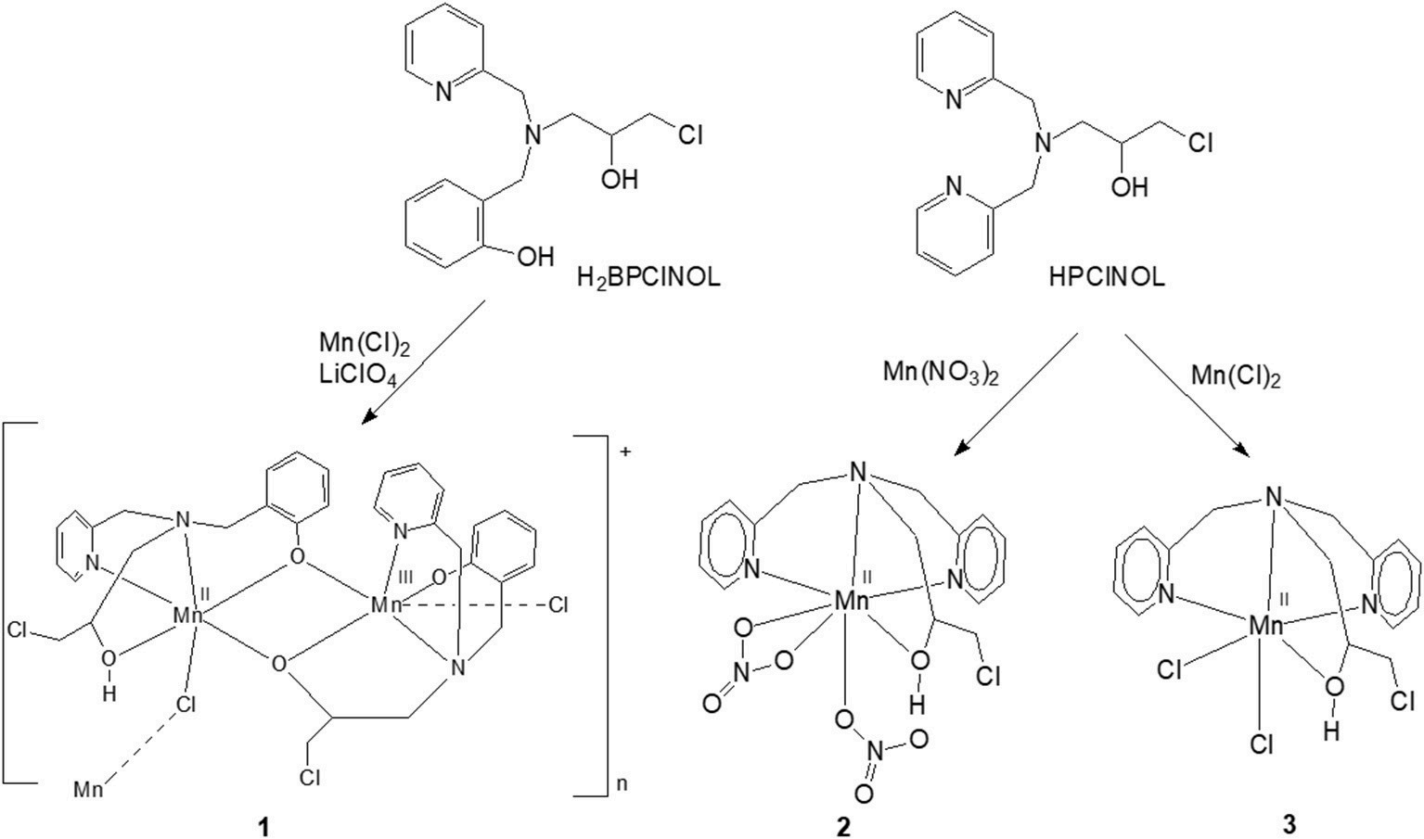
Scheme of synthesis of **1** and the related manganese complexes **2** and **3** described previously by our group; both have SOD/CAT activities. Only the monomeric unit of the cation **1** is shown (Lessa et al., 2007, 2009; Ribeiro et al., 2015).

Previously, we have described the synthesis of a tripodal tetradentade ligand HPClNOL = 1-(bispyridin-2-ylmethyl-amino)-3-chloropropan-2-ol (Horn et al., 2005a) and studied its coordination behavior with manganese(II) salts (Figure 1) (Lessa et al., 2007; Ribeiro et al., 2015). Their antioxidant properties have been also evaluated as a model for SOD and/or CAT enzymes (Lessa et al., 2009; Ribeiro et al., 2015). In an attempt to develop new and more active compounds with SOD/CAT activities, we employed a similar tripodal tetradentate ligand, i.e., H_2_BPClNOL = N-(2-hydroxybenzyl)-N-(2-pyridylmethyl)[(3-chloro)(2-hydroxy)] propylamine (Figure 1) (Horn et al., 2000), for the synthesis of a related manganese compound. Here, we present the properties of the new and rare polymeric mixed valence Mn(II)Mn(III) complex and the evaluation of its kinetic properties and mechanism of action with respect to its SOD and CAT mimetic activities.

## Experimental section

### Materials and methods

All chemicals and reagents were purchased from Sigma-Aldrich and used as such. UV-Vis, EPR, and MS investigations were carried out employing spectroscopic, HPLC or MS quality solvents. Dimethylsulfoxide (DMSO) was distilled over drying agents under an inert atmosphere, prior to EPR studies. It was stored over drying agents under inert atmosphere and transferred by syringe.

### Physical chemical characterization

Infrared spectra were recorded on a Shimadzu FT-IR 8300 spectrophotometer. The solid sample was prepared in a KBr pellet and the spectrum were recorded over the frequency range of 400–4,000 cm^−1^. UV-Vis spectra for the ligand and for the Mn complex were recorded in CH_3_CN on a UV-Vis Varian Cary 50 Bio spectrophotometer. The electrical conductivity of a 1 × 10^−3^ mol dm^−3^ CH_3_CN solution of **1** was measured with a Biocristal conductometer. Melting points were measured on a Microquimica MQAPF-301 apparatus. The purity of the complex was determined by combustion elemental analyses conducted with a Thermo Scientific FLASH 2000 CHNS/O analyzer. Full scan mass spectra were obtained on a MicroTOF LC Bruker Daltonics spectrometer equipped with an electrospray source operating in positive ion mode. Samples were dissolved in a CH_3_CN/H_2_O (50/50) solution and were injected in the apparatus by direct infusion. Theoretical isotopic patterns were calculated using the software ESI Compass 1.3 for micrOTOF, DataAnalysis version 4.0 SP 1 from Bruker Daltonik GmbH. EPR spectra were recorded on a Bruker Elexsys E500 EPR spectrometer equipped with a Bruker ER036TM Teslameter and frequency counter for calibration of the magnetic field and microwave frequency, respectively. Low temperature (140 K) at the sample position employed a nitrogen flow-through system in conjunction with a liquid nitrogen Eurotherm ER4131vt temperature controller. Computer simulation of the dimanganese EPR spectra employed Molecular Sophe28 in conjunction with Octave29 to optimize the spin Hamiltonian parameters. The magnetic data were collected using an MPMS-XL 5T (Quantum Design) SQUID magnetometer. Sample preparation involved pressing the powder into PTFE tape to prevent field-induced reorientation. Data were corrected for diamagnetic contributions from the sample using Pascal's constants, and from the sample holder. Effective magnetic moments were calculated using the relationship μ_eff_ = 2.828(χ_M_T)${}^{\frac{1}{2}}$.

### Ligand and complex syntheses

The ligand H_2_BPClNOL was synthesized by a reaction between the secondary amine N-(2-hydroxybenzyl)-N-(pyridin-2-ylmethyl)amine (HBPA) and epichlorohydrin, as reported previously (Horn et al., 2000). The complex ([(HBPClNOL)Mn(II)Mn(III)(BPClNOL)(Cl)](ClO_4_)·2H_2_O)_n_, **1** (Figure 1), was prepared in a reaction between H_2_BPClNOL (1.0 mmol, 0.31 g), dissolved in 10 cm^3^ of propan-2-ol and a solution containing MnCl_2_·4H_2_O (1.0 mmol, 0.20 g) and LiClO_4_ (1 mmol, 0.11 g), by refluxing over 1 h. After allowing the brown solution to stand for a few days, a crystalline brown solid was filtered off, washed with ethyl ether and dried under vacuum. After removing the crystals, the slow evaporation of the solvent resulted in the formation of an unidentified oily material. Yield: 0.20 g (22%). m.p. 243°C. IR (cm^−1^): ν(OH), 3422–3483 (s); ν(CH), 3,067 and 3,030 (s); ν(CH_2_), 2,969(s); ν(CH_2_), 2,924 (s); (C = C and C = N), 1,601 (s), 1,574 (s), 1,478 (s) and 1,456 (s); ν(ClO${}_{4}^{-}$), 1,121 and 1,020 (s); γ (CH), 758 (s) and 775 (s). Anal. calcd for [(HBPClNOL)Mn(II)Mn(III)(BPClNOL)(Cl)](ClO_4_)2H_2_O (C_32_H_39_Cl_4_Mn_2_N_4_O_10_, MW = 891.37 g mol^−1^): C, 43.12; H, 4.41; N, 6.29. Found: C, 42.77; H, 4.01; N, 5.92%. Ω = 123 μS cm^−1^ (1:1 electrolyte, CH_3_CN).

### X-Ray crystallography

The single crystal X-ray diffraction data of complex **1** were collected at 150(2)K on a Bruker D8 Venture diffractometer equipped with Photon 100 CMOS detector and using MoKα radiation (0.71073 Å) from an INCOATEC micro-focus source. Final lattice parameter values and integrated intensities were obtained using SAINT software (SAINT, 2015), and a multi-scan absorption correction was applied with SADABS (Krause et al., 2015). The structure was solved by direct methods using intrinsic phasing implemented in SHELXT (Sheldrick, 2015). The model was refined applying the full-matrix least-squares method using SHELXL (Sheldrick, 2015). All non-hydrogen atoms were refined with anisotropic displacement parameters. Hydrogen atoms were placed at calculated positions and refined using a riding model.

### Catalase-like activity

Catalase activity was measured by employing two different methods: (i) the decrease of H_2_O_2_ concentration was followed by UV-Vis spectroscopy at 240 nm (Beers and Sizer, 1952), and (ii) the rate of O_2_ production was measured employing a Clark-type electrode from Hansatech Instruments, model Oxygraph+.

The concentration of the H_2_O_2_ was previously determined by iodide/thiosulfate titration according to the literature (Ribeiro et al., 2009). All the reactions between complex **1** and H_2_O_2_ were performed in buffered and unbuffered solutions, as well as in the presence of piperazine. For the studies in unbuffered water solutions, 0.2 cm^3^ of an aqueous solution of complex **1** (2.5 × 10^−4^ mol dm^−3^) was added to a cuvette, followed by the addition of a certain amount (dependent on the required concentration) of water and H_2_O_2_ to reach a final volume of 2.2 cm^3^. Subsequently, the decrease in the absorption of the band attributed to H_2_O_2_ was monitored in a 1 cm path length cell. A similar study was performed using a buffered system (phosphate buffer, 0.05 mol dm^−3^, pH 7.8). For the study with piperazine, 0.2 cm^3^ of an aqueous solution of complex **1** (2.5 × 10^−4^ mol dm^−3^) was added to a cuvette, followed by the addition of 0.1 cm^3^ of an aqueous solution of piperazine (0.1 mol dm^−3^) and appropriate amounts of water and H_2_O_2_ to reach a final volume of 2.2 cm^3^. In this solution, the final concentration of **1** was 2.27 × 10^−5^ mol dm^−3^, while that of the piperazine was 4.54 × 10^−3^ mol dm^−3^, with a resulting pH of 9.73. The experiments were always carried out at 25°C. The consumption of H_2_O_2_ was again monitored spectrophotometrically as described above. These measurements were performed in triplicates and the kinetic parameters (i.e., K_M_, k_cat_, k_cat_/K_M_) were determined from a fit of the data to the Michaelis-Menten equation. Using the Clark electrode, the O_2_ production was followed for 120 s, but the rate of the reaction was measured during the first 50 s. The concentrations of **1** and piperazine were the same as described for the UV-Vis experiments.

The reaction was also investigated by EPR. A fresh solution of **1** was prepared in CH_3_CN (1.10^−3^ mol dm^−3^). From this solution 0.200 cm^3^ was placed in an EPR tube, which was then frozen at 140 K and the EPR spectrum recorded. The tube was then allowed to thaw at room temperature (~15 min). To this solution, in the EPR tube, 50 mm^3^ of an aqueous solution of piperazine (0.1 mol dm^−3^) was added. The solution was frozen again and the EPR spectrum recorded. Subsequently, the sample was allowed to thaw again (~15 min), and 50 mm^3^ of an aqueous solution of H_2_O_2_ (0.1 mol dm^−3^) was added. After freezing, another EPR spectrum was recorded. This study was repeated in duplicates.

All these experiments were carried out using crystalline samples that contains a mixture of two diastereomers (see x-ray section for more details).

### SOD-Like activity

The SOD activity of complex **1** was assessed employing the nitroblue tetrazolium (NBT) method, using xanthine/xanthine oxidase as a source of the superoxide anion, as described previously (Ribeiro et al., 2015). The kinetic studies were carried out in phosphate buffer (pH = 7.8). Stock solutions of xanthine (4.5 × 10^−4^ mol dm^−3^), NBT (5.6 × 10^−5^ mol dm^−3^) and xanthine oxidase (0.2 U cm^−3^), all purchased from Sigma-Aldrich, were prepared using phosphate buffer. In a control (blank) experiment the stock solutions of xanthine (1 cm^3^) and NBT (1 cm^3^) were mixed with phosphate buffer (0.4 cm^3^), and, at the end, xanthine oxidase (0.20 cm^3^) was added to the cuvette. To evaluate the SOD activity of **1**, different concentrations of the complex were added to the cuvette: 9.62 × 10^−8^, 1.92 × 10^−7^, 3.85 × 10^−7^, 5.77 × 10^−7^, and 7.69 × 10^−7^ mol dm^−3^.

The obtained IC_50_ was transformed to k_cat_ employing the equation proposed by McCord and Fridovich, k_cat_ = k_NBT_ x [NBT]/IC_50_, where K_NBT_ = 5.94 x 10^4^ M^−1^ s^−1^ (Grau et al., 2014; Ledesma et al., 2015).

The SOD like activity of **1** was also studied by EPR. A solution containing superoxide anion radical was generated in DMSO using the procedure described previously (Valentine et al., 1984). Briefly, 7 mg of KO_2_ was stirred in 1 cm^3^ of dried DMSO, for 2 h, resulting in a pale yellow solution (0.1 mol dm^−3^). A fresh solution of **1** was prepared in dried DMSO (1.6 × 10^−3^ mol dm^−3^), resulting in a brown solution. The KO_2_ solution (0.200 cm^3^) was placed in an EPR tube and the solution frozen and the spectrum recorded. The tube was removed from the cavity and allowed to sit at room temperature until a pale yellow solution was obtained again. Then, 200 μL of the solution of complex **1** was added to the former, resulting in a color change from pale yellow to reddish brown. The solution was frozen and the spectrum recorded. To follow the changes in the intensity of the superoxide EPR signal, the tube was removed from the cavity and allowed to thaw at room temperature; this was repeated until no more changes in the intensity of the spectrum were observed. This study was repeated in duplicate. As a control, a similar reaction was carried out using a MnCl_2_·4H_2_O solution (1.6 × 10^−3^ mol dm^−3^).

All these experiments were carried out using crystalline samples that contain a mixture of two diastereomers (see x-ray section for more details).

## Results and discussion

### Syntheses

The ligand H_2_BPClNOL is a tripodal ligand with a N_2_O_2_ donor atom set and is able to form mono- and dinuclear complexes with different metal ions, including iron, nickel, copper and zinc (see below for more details about these complexes). Here, we report the first manganese complex obtained with this ligand. The reaction between H_2_BPClNOL and MnCl_2_·4H_2_O resulted in a new compound that was isolated in the form of brownish crystals suitable for X-ray diffraction. The X-ray data have revealed (see below) the presence of an unusual, one dimensional mixed-valence Mn(II)Mn(III) chain. The elemental analysis agrees with the X-ray data, indicating high purity of the prepared compound. This complex is stable in air, in the solid state and in CH_3_CN solution. Its solution shows a brownish color, suggesting the presence of manganese in oxidation state higher than +2. This indicates that H_2_BPClNOL shows a different behavior when compared with HPClNOL, which forms mononuclear Mn(II) complexes (Figure 1).

### X-Ray molecular structure

**Figure 2:**
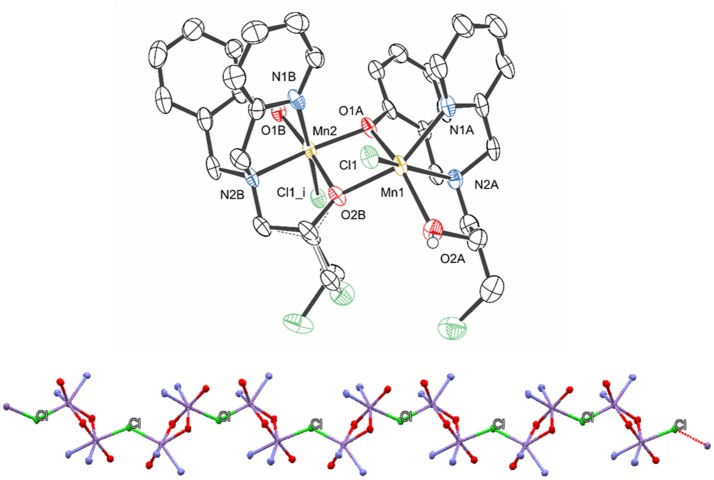
Structure representation of compound **1** (hydrogen atoms, perchlorate anion, and water of crystallyzation were omitted for clarity) **(Top)** and view of the polymeric chain **(Bottom)**, highlighting the chloro bridges that connect the monomers (only the atoms coordinated to the manganese centers are shown for clarity). The ellipsoids are drawn at 50% probability.

The molecular structure of **1** was solved by X-ray diffraction and the crystallographic data are presented in Tables 1 and 2. The data reveal the formation of a chain (Figure 2), where each subunit contains a heterovalent dimanganese (II/III) core, two molecules of the ligand and one chloro ligand, resulting in the composition [Mn(II)Mn(III)(HBPClNOL)(BPClNOL)Cl]^+^, where HBPClNOL and BPClNOL stand for the mono- and dianionic form of H_2_BPClNOL, respectively. The monomers are connected through chloro bridges, which are asymmetrically bound to the manganese centers [Mn1-Cl1 = 2.4908 (17), Mn2-Cl1_i_ = 2.6162(18) Å]. As shown in Figure 1, the ligand H_2_BPClNOL has two oxygen (phenol and alcohol) and two nitrogen (pyridine and tertiary amine) atoms as coordinating groups; interesting is the fact that the two molecules of the ligand coordinate differently to the metal centers, mainly with respect to their phenol and alcohol groups (see Figure 1). For a better explanation of the molecular structure of complex **1**, we label the two molecules of the ligand present in this complex as A and B in the X-ray structure representation (Figure 2). Ligand A (monoanion) shows a tetradentate coordination mode in which the phenol is acting as a bridging group [Mn1-O1A = 2.249(4) and Mn2-O1A = 1.934(4) Å] and the alcohol group is protonated and acts as a terminal ligand [Mn1-O2A = 2.201(5) Å]. On the other hand, in ligand B (dianion), the alcohol is deprotonated and acting as a bridging group [Mn2-O2B = 1.899(4), Mn1-O2B = 2.129(4)Å], while the phenol group is deprotonated as well but coordinating as a non-bridging ligand only to Mn2 [Mn2-O1B = 1.892(5) Å]. Furthermore, the carbon atom of the alcohol group is chiral and two isomers are present in the compound. In ligand A, the R isomer is observed, while the S isomer is seen in ligand B. It is important to note that compound **1** crystallizes in the centrosymmetric space group P2_1_/n. Due to the relation of symmetry associated with this space group, the crystal also shows molecules in which the isomers are opposite to those observed in the molecule shown in Figure 2. Molecules showing two chiral centers can form four diastereomers, which can be identified as RR, SS, RS, and SR. The x-ray data revealed that only two of them were formed, the RS and SR. Although the RR and SS were foreseen, they were not present in the crystals evaluated by x-ray analyses, even when the crystals were obtained from different syntheses. It is possible that the RR and SS diatereomers did not crystallize togheter with the RS and SR species, since they can result in compounds with different solubility, or that the dinuclear species are not formed due to steric hindrance. This can be one reason to explain the low yield observed in the synthesis. We hope to address this behavior in a future work.

**Table 1:** Crystal data and structure refinement details for complex **1**.

**Empirical formula**	**[Mn_2_(C_16_H_17_ClN_2_O_2_)(C_16_H_18_ClN_2_O_2_)Cl]ClO_4_·1.67(H_2_O)**
Formula weight	891.35
Temperature/K	150.15
Crystal system	Monoclinic
Space group	P2_1_/n
a/Å	14.2991(15)
b/Å	12.4755(12)
c/Å	21.676(2)
α/°	90
β/°	103.558(5)
γ/°	90
Volume/Å^3^	3759.0(7)
Z	4
ρ_calc_g/cm^3^	1.575
μ/mm^−1^	1.016
F(000)	1828.0
Crystal size/mm^3^	0.29 × 0.137 × 0.115
Radiation	MoKα (λ = 0.71073)
2Θ range for data collection/°	4.388 to 50.7
Index ranges	−17 ≤ h ≤ 16,−14 ≤ k ≤ 15,−17 ≤ l ≤ 26
Reflections collected	24450
Independent reflections	6847 [R_int_ = 0.0958, R_sigma_ = 0.1010]
Data/restraints/parameters	6847/19/498
Goodness-of-fit on F^2^	1.023
Final R indexes [I ≥2σ (I)]	R_1_ = 0.0753, wR_2_ = 0.1747
Final R indexes [all data]	R_1_ = 0.1271, wR_2_ = 0.2087
Largest diff. peak/hole / e Å^−3^	0.99/-1.11

**Table 2:** Selected bonds distances (Å) and angles (deg) for complex **1**.

**Mn1—O1A**	**2.249(4)**	**Mn2—O1B**	**1.892(5)**
Mn1—O2A	2.201(5)	Mn2—O2B	1.899(4)
Mn1—N1A	2.240(5)	Mn2—N1B	2.263(5)
Mn1—N2A	2.314(5)	Mn2—N2B	2.073(5)
Mn1—O2B	2.129(4)	Mn2—O1A	1.934(4)
Mn1—Cl1	2.4908(17)	Mn2—Cl1^(i)^	2.6162(18)
Mn1—Mn2	3.1593(14)		
O2B—Mn1—O2A	95.50(17)	O1B—Mn2—O2B	172.81(17)
O2B—Mn1—N1A	153.49(19)	O1B—Mn2—O1A	103.45(18)
O2A—Mn1—N1A	111.01(19)	O2B—Mn2—O1A	82.53(17)
O2B—Mn1—O1A	70.50(14)	O1B—Mn2—N2B	93.3(2)
O2A—Mn1—O1A	148.14(17)	O2B—Mn2—N2B	81.9(2)
N1A—Mn1—O1A	86.07(17)	O1A—Mn2—N2B	158.3(2)
O2B—Mn1—N2A	112.75(18)	O1B—Mn2—N1B	87.18(19)
O2A—Mn1—N2A	75.43(18)	O2B—Mn2—N1B	97.12(19)
N1A—Mn1—N2A	75.44(19)	O1A—Mn2—N1B	87.82(18)
O1A—Mn1—N2A	83.74(18)	N2B—Mn2—N1B	79.22(19)
O2B—Mn1—Cl1	88.78(12)	O1B—Mn2—Cl1^(i)^	87.15(14)
O2A—Mn1—Cl1	85.96(13)	O2B—Mn2—Cl1^(i)^	88.45(14)
N1A—Mn1—Cl1	93.05(15)	O1A—Mn2—Cl1^(i)^	94.21(14)
O1A—Mn1—Cl1	120.95(13)	N2B—Mn2—Cl1^(i)^	100.39(15)
N2A—Mn1—Cl1	152.45(15)	N1B—Mn2—Cl1^(i)^	174.28(15)
Mn1—O1A—Mn2	97.84(2)	Mn1—O2B—Mn2	103.21(2)

Symmetry ^codes:^^(i)^^−x+3/2^, ^y−1/2^, ^−z+1/2;^^(ii)^^−x+3/2^, ^y+1/2^, ^−z+1/2^

The averaged bond lengths around the two metal ions are 2.27 and 2.11 Å for the Mn1 and Mn2 ions, respectively. Based on (i) the bond distances, (ii) the fact that the Mn(III) ion has a smaller ionic radius than Mn(II) (Gelasco et al., 1997; Singh et al., 2015), (iii) the asymmetric coordination of the bridging chloride, and (iv) the fact that the dianionic form of H_2_BPClNOL is a harder Lewis base than the monoanionic one, it is plausible to assume that Mn1 and Mn2 are in the +2 and +3 oxidation states, respectively. Furthermore, the Mn1-Cl1 bond lengths are similar to those observed in other complexes containing Mn(II)-Cl bonds (2.425–2.472 Å) (Reddig et al., 2004). The Mn^···^Mn distance is 3.1593(14) Å, which is significantly shorter than the distances observed for a series of dinuclear Mn(II) complexes containing derivatives of the ligand 2-{[bis(pyridin-2-ylmethyl)amino]-methyl}phenol [3.392(8) to 3.493(2) Å] (Reddig et al., 2004). Furthermore, for a family of complexes containing the ligand 1,3-bis(salicylideneamino)-2-propanol, with which dinuclear Mn(II)Mn(II), Mn(II)Mn(III), Mn(III)Mn(III) and Mn(III)Mn(IV) complexes with di-μ-alkoxide bridges were generated, the Mn^···^Mn distance is in the range between 3.25 and 3.33 Å (Gelasco et al., 1997).

Several structures containing H_2_BPClNOL and other metal ions have been described in the literature. This ligand forms a dinuclear complex with Cu(II), containing di-chloro bridges. However, mononuclear Cu(II), Zn(II), and Fe(III) complexes have also been observed (Fernandes et al., 2010; Gomes et al., 2017). Interestingly, in the presence of Ni(II), a dinuclear species containing two phenoxide bridges was formed (Horn et al., 2006a), while the structures of three diiron(III) complexes demonstrate the presence of alkoxide bridges (Horn et al., 2005b, 2006b). In contrast, the mixed valence +2/+3 dinuclear Mn species described here has mixed bridging groups (alkoxide and phenoxide moieties). It appears that the oxidation state of the metal ions is a determining factor for the identity of the bridging groups [i.e., oxidation state-dependent isomerism (Mitić et al., 2003)]. Thus, the homovalent +3/+3 (iron complexes) and +2/+2 (nickel complex) systems have dialkoxide and diphenoxide bridges, respectively, while the heterovalent +2/+3 systems have an alkoxide and a phenoxide bridge.

### Infrared, UV-VIS, ESI-(+)-MS, and EPR characterization

The IR spectrum of the Mn(III)Mn(II) complex **1** was recorded and compared with that of its free ligand H_2_BPClNOL in the region between 4,000 and 400 cm^−1^. For H_2_BPClNOL, characteristic bands of the aromatic group are observed at 1,595, 1,558, 1,475, and 1,433 cm^−1^, assigned to ν C = N and ν C = C. For complex **1**, the corresponding bands are observed at 1,601, 1,574, 1,478, and 1,456 cm^−1^. H_2_BPClNOL also shows an intense band at 1,289 cm^−1^ that is attributed to ν C-O of the phenol group; the corresponding feature is observed at 1,275 cm^−1^ in complex **1**. Furthermore, **1** has two intense bands at 1,121 and 1,080 cm^−1^, which are associated with the perchlorate anion. These bands are absent in the spectrum of the ligand.

The electronic spectrum in acetonitrile of complex **1** is dominated by intense bands in the UV range: 238 nm (ε = 1.8 × 10^4^ dm^3^ mol^−1^ cm^−1^), 262 nm (ε = 1.5 × 10^4^ dm^3^ mol^−1^ cm^−1^), 316 nm (ε = 4.7 × 10^4^ dm^3^ mol^−1^ cm^−1^) and 364 nm (ε = 3.3 × 10^4^ dm^3^ mol^−1^ cm^−1^). In the Vis range, a shoulder is observed at 459 nm (3.0 × 10^3^ dm^3^ mol^−1^ cm^−1^). While the UV bands are attributed to π → π^*^ intraligand transitions, the lower energy transition is assigned to a phenolate → Mn^III^ LMCT transition (Karsten et al., 2002; Singh et al., 2015).

The analysis of a solution containing complex **1** by ESI-(+)-MS indicated the presence of peaks with *m/z* of 201, 307, 359, 377, 419, 665, 718, 735, 754, and 763. The peaks at *m/z* 307 and 201 are ascribed to the protonated form of the ligand and to its fragment, respectively. The peak at *m/z* 665 is ascribed to a mononuclear cation containing two molecules of H_2_BPClNOL (herewith referred to as H_2_L): [Mn(III)(HL)_2_]^+^. The peaks with *m/z* 718, 735, 754 and 763 are ascribed to [Mn(III)Mn(II)(L)_2_]^+^, [Mn_2_(III)(L)_2_(OH)]^+^, [Mn(III)Mn(II)(HL)(L)(Cl)]^+^, [Mn_2_(III)(L)_2_(CN)(H_2_O)]^+^, respectively. These proposed assignments are based on the comparison of the simulated and experimental isotopic pattern and on the MS/MS data for each peak (see Figures ESI1–9 in Supplementary Material). MS/MS data indicate that the cations with *m/z* 763, 754, and 735 yield the cation with *m/z* 718, which corresponds to a dinuclear Mn(III)Mn(II) arrangement, in agreement with the data obtained from x-ray diffraction. It should be pointed out that the species with *m/z* 718 and the one with *m/z* 754 both agree with the presence of mixed valence Mn centers. In particular, the species associated with *m/z* 754 is in perfect agreement with the molecular structure observed for the monomeric unit, as revealed by x-ray diffraction. A proposal for the structure of the main signals observed in the ESI-(+)-MS study is presented as suplementary information.

**Figure 3:**
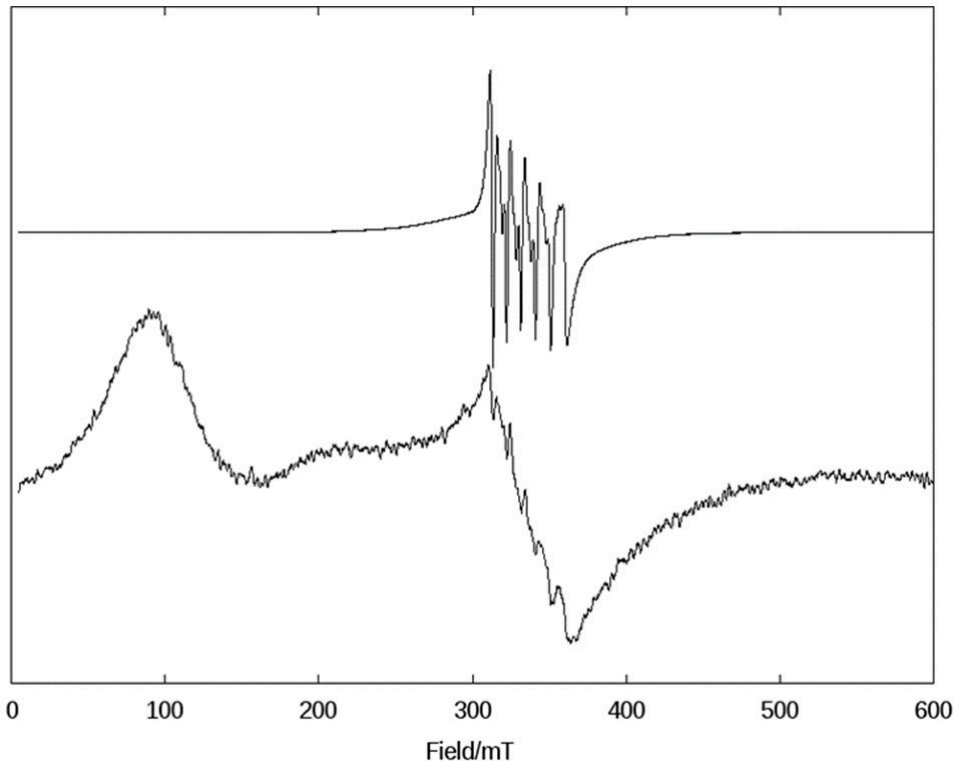
X-band CW EPR spectra of **1** in frozen solutions of DMSO **(Top)** and CH_3_CN **(Bottom)** at 140 K.

Due to the novelty of the mixed-valent, mixed-bridged and polymeric structure of **1** in the solid state, the effect of CH_3_CN, DMSO, and H_2_O on the molecular arrangement was investigated by EPR at 1.8 K (Figure ESI10) and 140 K (Figure 3) in order to probe if solvents promote structural changes. While the spectra recorded in H_2_O and DMSO are similar, they differ significantly from the spectrum in CH_3_CN, indicating that the solvent has a considerable effect on the structure of the compound.

In the solid state, compound **1** shows only one broad band around g = 2 (see Figure ESI10), but when measured in a CH_3_CN solution a six-line signal at g ~2, which is characteristic of Mn(II) ions, and a broad band at g ~7 are observed, indicating a significant change in the magnetic behavior of the system after solubilization. Broad resonances at low field have been previously described for coupled Mn(II)Mn(III) systems, and were interpreted in terms of the presence of ferro- or antiferromagnetically coupled Mn(II)Mn(III) cores. A feature of this low field signal is that for an antiferromagnetically coupled system, the signal disappears when the temperature decreases (Smith et al., 2009). On the other hand, in ferromagnetically-coupled Mn(II)Mn(III) dimers, the signal grows at low temperatures (Gelasco et al., 1997). We have observed that the signal around g ~7 increases upon lowering the temperature from 140 K to 1.8 K (Figure ESI11), which indicates that complex **1** contains a ferromagnetically-coupled Mn(II)Mn(III) dimer. This interpretation was further confirmed by magnetic measurements (see below). In addition, in Mn(II)Mn(III) systems with antiferromagnetic coupling, multiline features with as many as 36 lines can be observed around g = 2 due to the population of the S = $\frac{1}{2}$ state of the dinuclear manganese system (Smith et al., 2009; Sano et al., 2013; Jung and Rentschler, 2015; Magherusan et al., 2018). In contrast, for ferromagnetically-coupled Mn(II)Mn(III) complexes published EPR data vary, including compounds that only show a signal at low field (g >5), or only a signal at high field (g ~ 2), or a combination of both features (Schake et al., 1991; Gelasco et al., 1997; Rane et al., 2000). Thus, the spectral features of compound **1** are in agreement with other ferromagnetically-coupled Mn(II)Mn(III) systems, and the difference between the spectra in the solid state and in the CH_3_CN solution is ascribed to the dissociation of the polymeric structure in solution, leaving the dinuclear antiferromagnetically-coupled Mn(II)Mn(III) system.

In DMSO (and H_2_O) the EPR spectrum features six sharp lines (due to a ^55^Mn hyperfine intetraction, *I* = 5/2), typical of an isolated Mn(II) species and very similar to those obtained for the mononuclear complex [Mn(II)(HPClNOL)(NO_3_)_2_], **2** (Figure 1) (Lessa et al., 2009). HPClNOL is similar to H_2_BPClNOL, the ligand employed in this study, but has two pyridine groups instead of one pyridine and one phenol group (Figure 1). The same behavior was observed in aqueous solution. This observation suggests that the dinuclear structure of the monomer is not stable in DMSO and water and, therefore, only the six-line signal typical of isolated Mn(II) centers was observed (Lessa et al., 2009). In contrast, in acetonitrile, the dimeric structure is stable, resulting in a decrease in resolution and intensity of the features associated with the Mn(II) center.

### Magnetism

**Figure 4:**
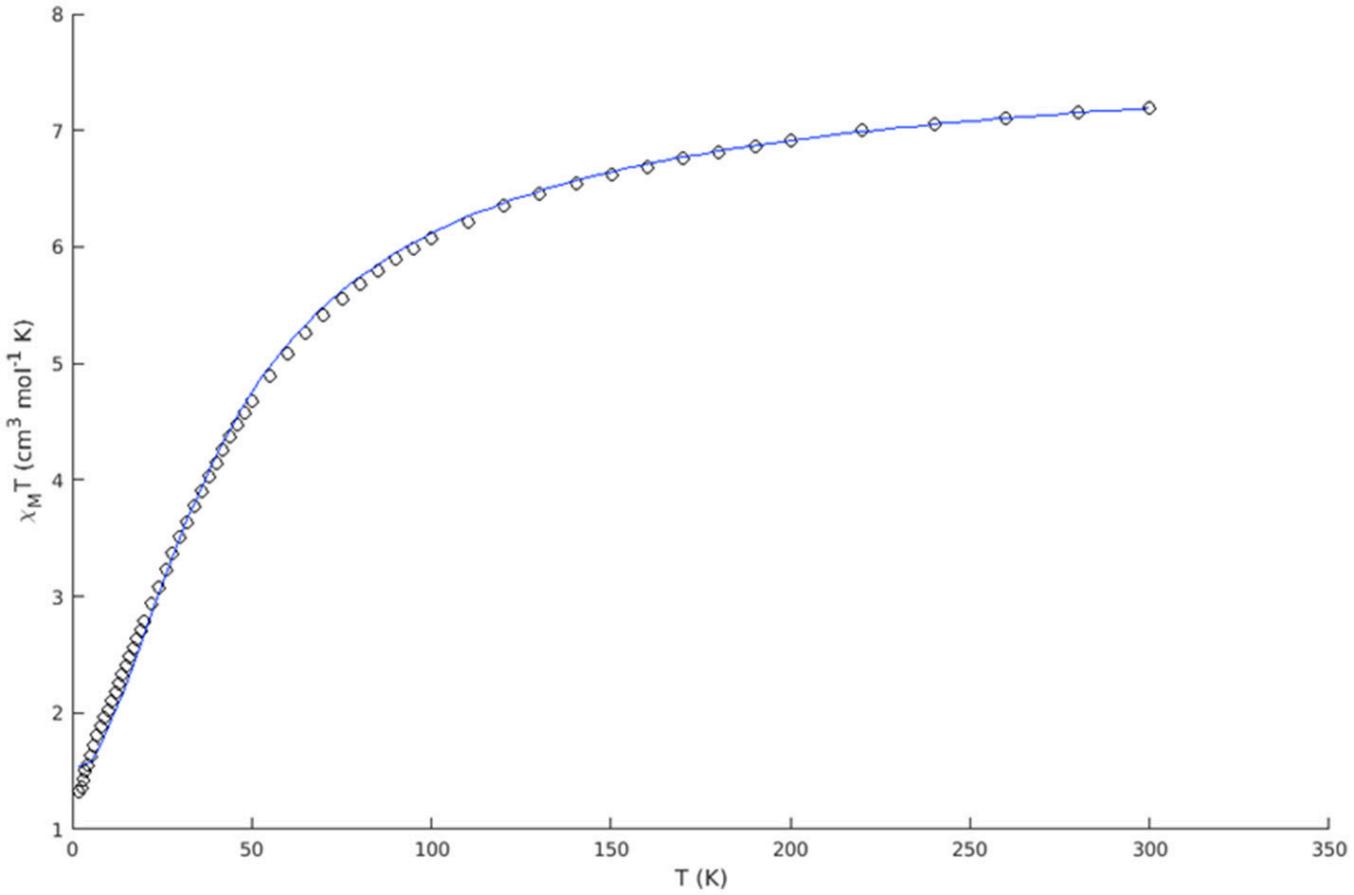
Experimental χ_M_T vs. T plot of complex **1** (open circles) and best fit (blue solid line).

The magnetic susceptibility of complex **1** was measured over the temperature range 2–300 K at 0.05 T. The experimental data are presented as a χ_M_T vs. T plot (Figure 4) of the Mn^II^Mn^III^ dinuclear unit.

The room temperature value of 7.20 cm^3^ mol^−1^ K (μ = 7.59 BM) is slightly lower than the theoretical value for two non-interacting spin systems of 7.38 cm^3^ mol^−1^ K (μ = 7.68 BM *g* = 2, *S*_A_ = 5/2, *S*_B_ = 2). The susceptibility steadily decreases with decreasing temperature, indicating antiferromagnetic interactions between the two metal centers. The low temperature value of 1.32 cm^3^ mol^−1^ K (μ = 3.25 BM) is higher than the low temperature limit (χ_M_T = 0.38 cm^3^ mol^−1^ K, μ = 1.73 BM *g* = 2, *S* = 1/2) of an antiferromagnetically coupled system of this kind. In a simple dinuclear complex, this would indicate the presence of mononuclear impurities, however, the X-ray crystal structure indicates that the dinuclear Mn^II^(μ-OR)_2_Mn^III^ units are bridged by a chloride ion to form a one dimesion chain. The compound thus has a chain structure with alternating *S* = 2: *S* = 5/2 spin carriers and (μ-OR)_2_: μ-Cl interaction pathways. For this reason, attempts to fit the data for a single coupling constant were unsatisfactory, and we considered the Heisenberg chain Hamiltonian instead. The spin Hamiltonian in zero field is:

$$\begin{array}{l} \text{H}=-J\underset{i}{\sum }{\text{S}}_{B_{i}}\left[\left(1+\alpha \right){\text{S}}_{A_{i}}+\left(1-\alpha \right){\text{S}}_{A_{i+1}}\right] \end{array}$$

The derivation of the function for an alternating ferromagnetic chain compound was described by Pei (Pei et al., 1988), where χ_M_T is defined as:

$$\begin{array}{l} \chi_{M}T=\frac{N\beta^{2}}{3k}\frac{g^{2}\left[s\left(s+1\right)\left(1-P\right)+2QR\right]+2gG\left(Q+R\right)+G^{2}\left(1+P\right)}{1-P} \end{array}$$

with

$G=g_{A}{\left[S_{A}\left(S_{A}+1\right)\right]}^{1/2}g=g_{B}s=S_{B}x=J/kT$

$$P=\frac{A_{1}}{A_{0}}$$

$$Q=\frac{x\left[\left(1+\alpha \right)B_{0}+\left(1-\alpha \right)B_{1}\right]}{A_{0}}$$

$$R=\frac{x\left[\left(1-\alpha \right)B_{0}+\left(1+\alpha \right)B_{1}\right]}{A_{0}}$$

$$A_{0}=\frac{2\pi }{{\text{Λ}}^{2}}\sum_{\sigma =-s}^{s}\sum_{\varepsilon =\pm }^{}\frac{\varepsilon \exp\left(\sigma \lambda_{\varepsilon }\right)}{\sigma^{2}}(\sigma \lambda_{\varepsilon }-1)$$

$$\begin{array}{l} A_{1}=\frac{\pi }{{\text{Λ}}^{2}}\sum_{\sigma =-s}^{s}\sum_{\varepsilon =\pm }^{}\frac{\varepsilon \exp\left(\sigma \lambda_{\varepsilon }\right)}{\sigma^{4}}[\sigma^{3}\lambda_{\varepsilon }^{3}-3\sigma^{2}\lambda_{\varepsilon }^{2}\\ \;+\left(6-\sigma^{2}\lambda^{2}\right)\sigma \lambda_{\varepsilon }+\sigma^{2}\lambda^{2}-6] \end{array}$$

$$B_{0}=\frac{2\pi }{{\text{Λ}}^{2}}\sum_{\sigma =-s}^{s}\sum_{\varepsilon =\pm }^{}\varepsilon \;exp(\sigma \lambda_{\varepsilon })$$

$$B_{1}=\frac{\pi }{{\text{Λ}}^{2}}\sum_{\sigma =-s}^{s}\sum_{\varepsilon =\pm }^{}\frac{\varepsilon \exp\left(\sigma \lambda_{\varepsilon }\right)}{\sigma^{2}}[\sigma^{2}\lambda_{\varepsilon }^{2}-2\sigma \lambda_{\varepsilon }+2-\sigma^{2}\lambda^{2}]$$

$$\lambda_{+}=-2x\;\lambda_{-}=\alpha \;\lambda_{+}\;\lambda^{2}=2x^{2}(1+\alpha^{2})\;\Lambda^{2}=x^{2}(1+\alpha^{2})$$

with *g*_A_ = *g*_B_ = 2.05, *S*_A_ = 5/2 and *S*_B_ = 2. The data were fit for α and *J*, where *J* is defined as:

$$\begin{array}{l} J=J_{AB}{\left[S_{A}\left(S_{A}+1\right)\right]}^{1/2} \end{array}$$

**Scheme 1:**
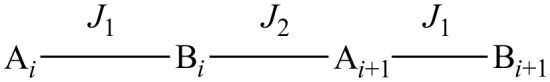
A representation of the exchange coupling constants (*J*_1_ and *J*_2_) of an alternating ferrimagnetic chain complex; A and B represent the two different spin carriers.

The two different coupling pathways (*J*_1_ and *J*_2_) are represented in Scheme 1 and the coupling constants are then given by:

$$\begin{array}{l} J_{1}=J_{AB}\left(1+\alpha \right)\;J_{2}=J_{AB}\left(1-\alpha \right) \end{array}$$

The best fit gave α = 1.029(2) and *J* = −7.614(40) cm^−1^, resulting in two exchange coupling constants of *J*_1_ = −5.224(13) and *J*_2_ = +0.076(13) cm^−1^. The antiferromagnetic coupling (*J*_1_) is ascribed to the interaction via the chloro bridge, where the more linear M-Cl-M angle of 129.34° is expected to facilitate antiferromagnetic interactions (Orchard, 2003). This conclusion is in agreement with other similar chloro bridged complexes (Fu et al., 1996; Gibson et al., 2003; Coates et al., 2010; Hirotsu et al., 2012; Zou et al., 2012).

The very weak ferromagnetic coupling (*J*_2_) is attributed to exchange via the di-OR bridge. The Mn-OR-Mn angles were found to be 97.84° and 103.21°, with a Mn-O-O-Mn torsion angle of 156.56°, which is consistent with ferromagnetic exchange (Gelasco et al., 1997; Wittick et al., 2004; Naiya et al., 2012; Hänninen et al., 2013). Similar structural features have also been observed in a set of di- and trinuclear mixed valence manganese complexes (Hänninen et al., 2013). The ferromagnetic coupling constants of the dinuclear complexes were found in the range of +2.15(6) to +7.9(7) cm^−1^, whereas a much smaller coupling constant of +0.04(7) cm^−1^ was observed in the case of one of the trinuclear complexes. The smaller value of *J* was attributed to a shift of the central Mn^II^ ion out of the plane of the bridging oxygens, reducing the ferromagnetic contribution to the coupling between the d*xy* and d*x*^2^-*y*^2^ orbitals. This distortion is not observed in the present case, and the small ferromagnetic coupling likely stems from the slightly elongated Mn-OR bond lengths of complex **1** (1.897–2.250 Å, average 2.052 Å) when compared to the reported dinuclear complexes (1.889–1.934 Å, average 1.912 Å).

The confirmation of the presence of ferromagnetic coupling involving the Mn(II)-(μ-OR)_2_-Mn(III) explains the behavior of the signal seen at *g* ~ 7 in the EPR spectrum, which does not disappear when the temperature drops from 140 to 1.8 K.

### Superoxide dismutase (SOD) activity

The SOD-like activity of complex **1** was studied employing the NBT assay in aqueous buffered solution (pH 7.8). NBT is a compound that undergoes reduction in the presence of superoxide anions, resulting in a purple species that may be monitored at 560 nm. The superoxide anions are generated at a constant rate by the xanthine/xanthine oxidase system (O'Connor et al., 2012). In this assay, the capability of the compound of interest (i.e., **1**) to prevent NBT reduction is evaluated. Thus, the concentration of the compound that inhibits 50% of NBT reduction corresponds to the IC_50_. As a control we determined that the pure ligand was not active. Relevant results are summarized in Table 3, together with corresponding data for other compounds, including the native SOD enzymes. The kinetic parameters (IC_50_ and *k*_cat_) related to the SOD-like activity of complex **1** are similar to those of complex **3** reported by us previously (see Table 3), and are in the same range observed for other manganese compounds.

**Table 3:** Kinetic parameters of reported manganese superoxide dismutase mimetics containing tripodal amine ligands and the natural enzyme.

**Compound**	**SOD Activity**		**References**
	**IC_50_ (μM)**	**10^6^ k_cat_ (M^−1^s ^−1^)**	
**1**^a^	0.370 ± 0.012	3.4	This work
**3** ^a^	0.34 ± 0.02	3.7	Ribeiro et al., 2015
[Mn(II)(TMIMA)_2_]^2+^^b^	1.6 ± 0.1	3.6	Durot et al., 2005
[Mn(II)(BMPG)(H_2_O)]^+b^	1.2 ± 0.5	4.8	Durot et al., 2005
[Mn(BIG)(H_2_O)_2_]^+b^	3.7 ± 0.6	1.5	Durot et al., 2005
[Mn(IPG)(MeOH)]^+b^	3.0 ± 0.6	1.9	Durot et al., 2005
[Mn(PBMPA)Cl(H_2_O)]^a/b^	2.67 ± 0.37	4.9	Pap et al., 2012
CuZn-SOD^a^	0.03	n.d	Weser et al., 1981
CuZn-SOD^c^	0.0026	n.d	Suksrichavalit et al., 2008
Human MnSOD^d^	–	800	Ramilo et al., 1999
*T. thermophiles*^e^	–	0.002	Bull et al., 1991

a study carried out with xanthine/xanthine oxidase-mediated reduction of NBT;

b *study carried out with xanthine/xanthine oxidase-mediated reduction of cytochrome c*;

c *study carried out with xanthine/xanthine oxidase SOD assay kit-WST*;

d *pulse radiolysis;*.

e *KO_2_. HPClNOL, 1-[bis(pyridin-2-ylmethyl)amino]-3-chloropropan-2-ol; TMIMA, tris[(1-methyl-2-imidazolyl)methyl]amine; BMPG, N,N-bis[(6-methyl-2-pyridyl)methyl]-glycinate; BIG, N,N-bis[(1-methyl-2-imidazolyl)methyl]glycinate; IPG, N-[(1-methyl-2-imidazolyl)methyl]-N-(2-pyridylmethyl)glycinate; PBMPA, N-propanoate-N,N-bis-(2-pyridylmethyl)amine*.

Attempts to evaluate the interaction between an aqueous solution of **1** and the superoxide anion produced by the xanthine/xanthine oxidase system by EPR, as published previously (dojindo.com)^1^, were unsuccessful. Therefore, although the SOD activity of **1** was measured in a buffered aqueous solution, we carried out an EPR investigation of the reaction in DMSO. In this context it is important to highlight that the EPR spectrum of **1** in water and in DMSO are identical, revealing the presence of mononuclear species.

**Figure 5:**
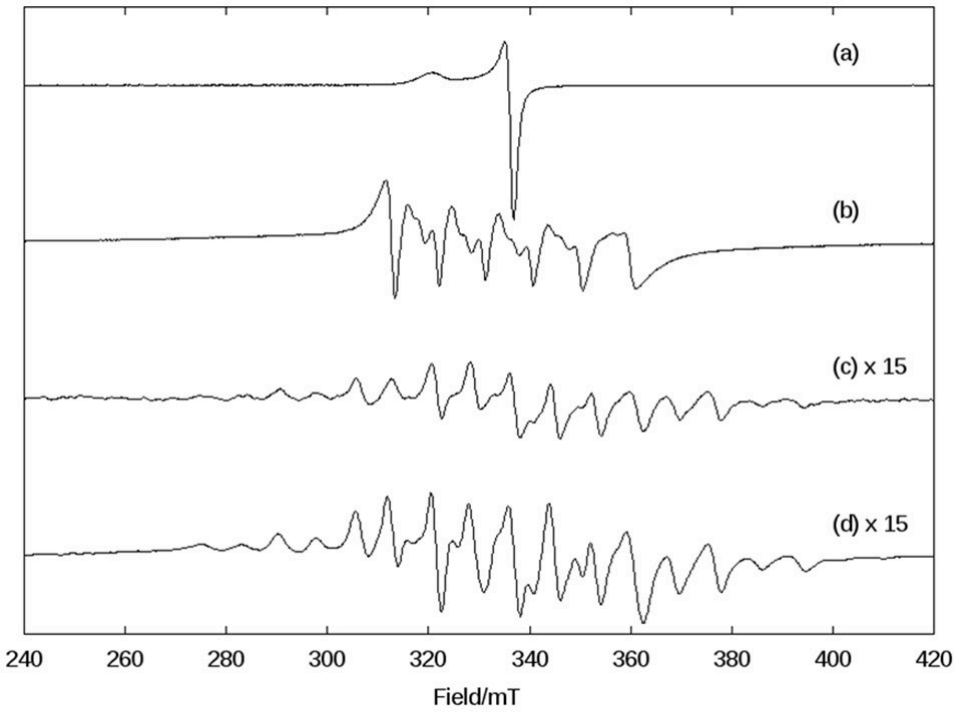
X-band CW EPR spectra in DMSO at 140 K of **(A)** superoxide (KO_2_), **(B)** complex **1** in dry DMSO, **(C)** the complex **1** immediately after the reaction with superoxide, and **(D)** the complex **1**, 1 h after the reaction with superoxide.

A DMSO solution of KO_2_ shows an anisotropic EPR spectrum (g_//_ = 2.11 and g_⊥_ = 2.01) characteristic of O${}_{2}^{•-}$ (Figure 5A) (Valentine et al., 1977). In dry DMSO the spectrum for complex **1** displays a six-line pattern, typical of isolated Mn(II) species as discussed above (Figures 3, 5B). Figure 5C shows the spectrum recorded immediately after the interaction between the superoxide anion and complex **1**. In this case, a 16-line feature is observed, which was previously ascribed to a Mn(III)Mn(IV) dimer containing an oxo bridge (Dubois et al., 2008; Jiang et al., 2009; Mitić et al., 2009). The simulation of this 16-line spectrum is shown in Figure ESI12, and is in excellent agreement with the experimental data. The simulation was performed employing the expression (Lessa et al., 2009):

$$\begin{array}{l} H=\beta B\cdot g\cdot S+\overset{2}{\underset{j=1}{\sum }}S\cdot A_{Mn}\cdot I_{Mn}-g_{n}\beta_{n}B\cdot I_{Mn} \end{array}$$

with the following *g* and *A*(Mn) matrices: *g*_x_ = 2.0014, *g*_y_ = 2.0030, *g*_z_ = 1.9865, *A*[Mn(III)]_1x_ = 136.3 × 10^−4^ cm^−1^, *A*[Mn(III)]_1y_ = 155.5 × 10^−4^ cm^−1^, *A*[Mn(III)]_1z_ = 103.4 × 10^−4^ cm^−1^, *A*[Mn(IV)]_2x_ = 75.3 × 10^−4^ cm^−1^, *A*[Mn(IV)]_2y_ = 68.6 × 10^−4^ cm^−1^, *A*[Mn(IV)]_2z_ = 77.4 × 10^−4^ cm^−1^. These spin Hamiltonian parameters are similar to those for other Mn(III)-(μ-O)-Mn(IV) species reported previously [Mn(III): *S* = 2, Mn(IV): *S* = 3/2, ground state *S* = 1/2; (Horner et al., 1999)].

As a control, we performed a reaction between a KO_2_ solution (DMSO) and Mn(Cl)_2_·4H_2_O. In this case, a different behavior was observed when compared to **1**. The signal associated with Mn(II) disappeared, suggesting that it underwent oxidation. Furthermore, the signal of the superoxide radical remained visible, indicating that MnCl_2_ did not promote the disproportionation of all superoxide molecules present in solution. On the other hand, compound **1** extinguished the EPR signal associated with the superoxide radical (see Figure ESI13).

Thus, using EPR spectroscopy we could demonstrate that in DMSO, compound **1** can decompose the superoxide anion, whose reaction pathway involves the formation of a dimanganese high-valent Mn(III)-oxo-Mn(IV) species, which is stable for at least 1 h (Figure 5D).

### Catalase (CAT) activity

**Figure 6:**
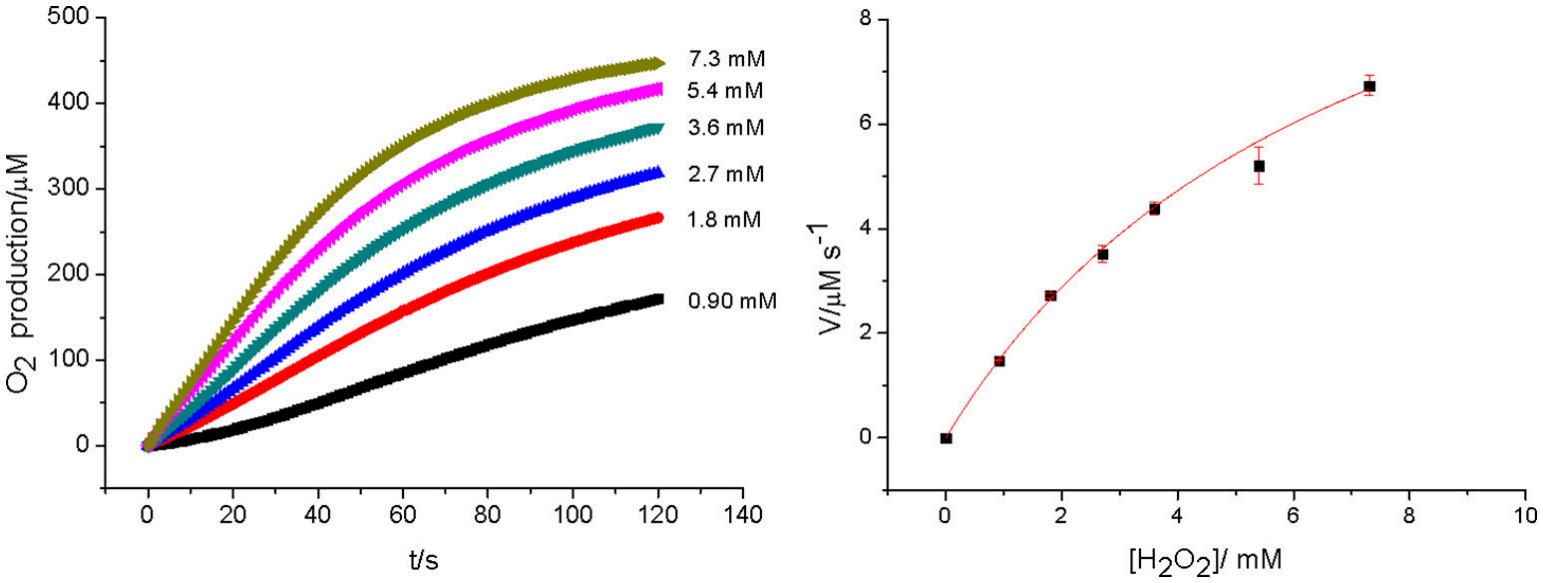
Rates of O_2_ production at [**1**] = 2.27 × 10^−5^ mol dm^−3^ and different concentration of H_2_O_2_ (left). The dependence of the rate on substrate concentration, together with a fit to the Michaelis–Menten equation (right).

Bacterial catalases from organisms such as *Lactobacillus plantarum, Thermus thermophiles*, or *Thermoleophilum album* (Whittaker, 2012) possess a dinuclear manganese cluster in their active sites. However, we also demonstrated that the mononuclear compounds **2** and **3** (Figure 1) have catalase activity (Lessa et al., 2009; Ribeiro et al., 2015). Since **1** in the solid state and in acetonitrile contains a dimanganese center, but forms a mononuclear complex in DMSO and H_2_O, it is plausible to assume that it may show CAT activity as well. We thus investigated the H_2_O_2_ disproportionation promoted by **1** under three different conditions. Firstly, the reaction was investigated in distilled water, but no activity was observed. Secondly, the reaction was performed in a buffered solution (phosphate buffer, 0.05 mol dm^−3^, pH 7.8), but again, no activity was observed. This behavior differs significantly from that observed for compounds **2** and **3**, which show CAT activity in pure water as well as buffered solutions (Lessa et al., 2009; Ribeiro et al., 2015). Thirdly, the assay was carried out with piperazine (0.1 mol dm^−3^, pH = 9.73) in an aqueous solution and bubbles were produced immediately after the addition of H_2_O_2_. Therefore, kinetic measurements were conducted in the presence of piperazine. The time course of O_2_ production at 25 °C in the presence of piperazine and at different concentrations of H_2_O_2_ is illustrated in Figure 6. The data were analyzed by a fit to the Michaelis-Menten equation. A similar study was performed by measuring the consumption of H_2_O_2_ by UV-Vis (see Figure ESI14). Relevant parameters are summarized in Table 4, together with corresponding data for other manganese compounds for comparison.

**Table 4:** Kinetic parameters of reported manganese catalase mimetics containing tripodal amine ligands and the natural enzyme.

**Compound**	**k_cat_ (s^−1^)**	**K_M_ (mM)**	**k_cat_/K_m_ (M^−1^ s^−1^)**	**Solvent system**	**References**
**1**^a^	2.59 ± 0.12	11.7 ± 1.1	221.3	H_2_O + piperazine	This work
**1**^b^	0.58 ± 0.03	7.23 ± 0.48	80.2	H_2_O + piperazine	This work
[Mn${}_{2}^{\text{II}}$(tpa)_2_(μ-Cl)_2_]^2+^	107	3.1	34,516	CH_3_CN	Shin et al., 2010
[Mn${}_{2}^{\text{II}}$(tpa)_2_(μ-Cl)_2_]^2+^	1.97	1.47	1,340	CH_3_CN + H_2_O	Shin et al., 2010
[Mn^II^(bpia)(μ-OAc)]${}_{2}^{2+}$	1,100	31.5	34,000	CONHCH_3_	Triller et al., 2002
[Mn_2_(L_1_-L_5_)_2_Cl_2_]	87.8–283	18–54.3	2,750–7,800	H_2_O/MeOH	Reddig et al., 2004
CAT(*T. thermophilus*)	2.6 × 10^5^	83	3.1 × 10^6^		Shank et al., 1994

a *Evaluated by UV-Vis spectroscopy by following H_2_O_2_ consuption*.

b *Measured by electrochemical O_2_ detection*.

*Tpa, (tris(2-pycolyl)amine); bpia, bis-(picolyl)(N-methylimidazol-2-yl)amine); L1-L_5_, derivatives of 2-{[bis(pyridin-2-ylmethyl)amino]methyl}phenol*.

The data presented in Table 4 reveal that the kinetic rates obtained for **1** are lower when O_2_ production is measured than when the consumption of H_2_O_2_ is recorded. This observation suggests that in the UV-Vis experiment, the change in the reading at 240 nm related to the H_2_O_2_ molecule may be influenced by changes in the absorption of **1** at this wavelength. Therefore, the UV-Vis method may not be suitable to evaluate the decomposition rate of H_2_O_2_ when in the presence of molecules that show intense absorption in a comparable wavelength range. Hence, we consider the kinetic parameters obtained with the Clark electrode as more reliable.

The kinetic data show that **1** is less active than other dinuclear manganese complexes containing tripodal ligands (tpa, bpia, L_1_-L_5_). This may be explained by the presence of piperazine, which can compete with H_2_O_2_ by the manganese coordination site. Furthermore, the presence of water has been considered as an inhibitor, too, as exemplified by the compound [Mn${}_{2}^{\text{II}}$(tpa)_2_(μ-Cl)_2_]^2+^, whose *k*_cat_ decreased around 50 times when the reaction was perfomed in CH_3_CN/H_2_O instead of anhydrous CH_3_CN. Thus, **1** shows a *k*_cat_ comparable to that of [Mn${}_{2}^{\text{II}}$(tpa)_2_(μ-Cl)_2_]^2+^.

**Figure 7:**
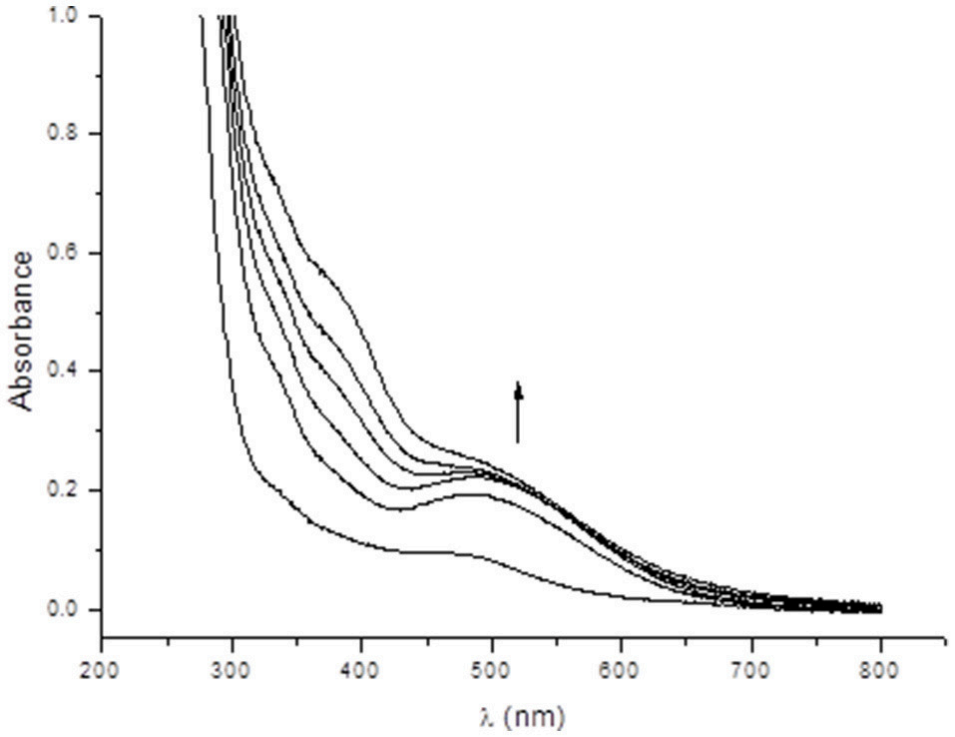
Electronic spectra showing the interaction between complex **1** and successive additions of 10 mm^3^ of a 0.1 mold m^−3^ aqueous piperazine solution, where the first spectrum refers to a solution of complex **1** in water (8 × 10^−5^ mol dm^−3^).

In an attempt to gain insight into the role of piperazine in the catalytic process, we investigated the interactions between complex **1** and piperazine with different techniques. In Figure 7 the electronic spectrum of **1** dissolved in water is shown as a function of an increasing amount of piperazine. Piperazine alone does not have any electronic transitions above 300 nm. However, two new distinct transitions were observed when this reagent was added to a solution containing complex **1**. The first transition (shoulder) at 459 nm, associated with a LMCT in complex **1** (*vide supra*), gained intensity and was red-shifted to ~500 nm. The increase in the intensity of this phenolate → Mn(III) LMCT as a function of piperazine concentration suggests that Mn(II) is undergoing oxidation. A second relevant band appears as a shoulder around 390 nm and is ascribed to an oxo Mn(III)/(IV) transition (Lessa et al., 2009). The driving force for the oxidative process may be linked to either the direct coordination of piperazine to the manganese ion or the deprotonation of the coordinating alcohol group from the ligand (which is protonated as seen in the molecular structure solved by x-ray diffraction). The spectral changes observed in Figure 7 also reveal the existence of two consecutive reactions. The band at 500 nm increases faster than the shoulder around 390 nm, supporting the hypothesis that the first step involves the oxidation induced by piperazine, resulting in an intermediate that reacts with O_2_. The final species contains a Mn(III)-oxo-Mn(IV) core, as evidenced by ESI-MS and EPR results (see below).

**Figure 8:**
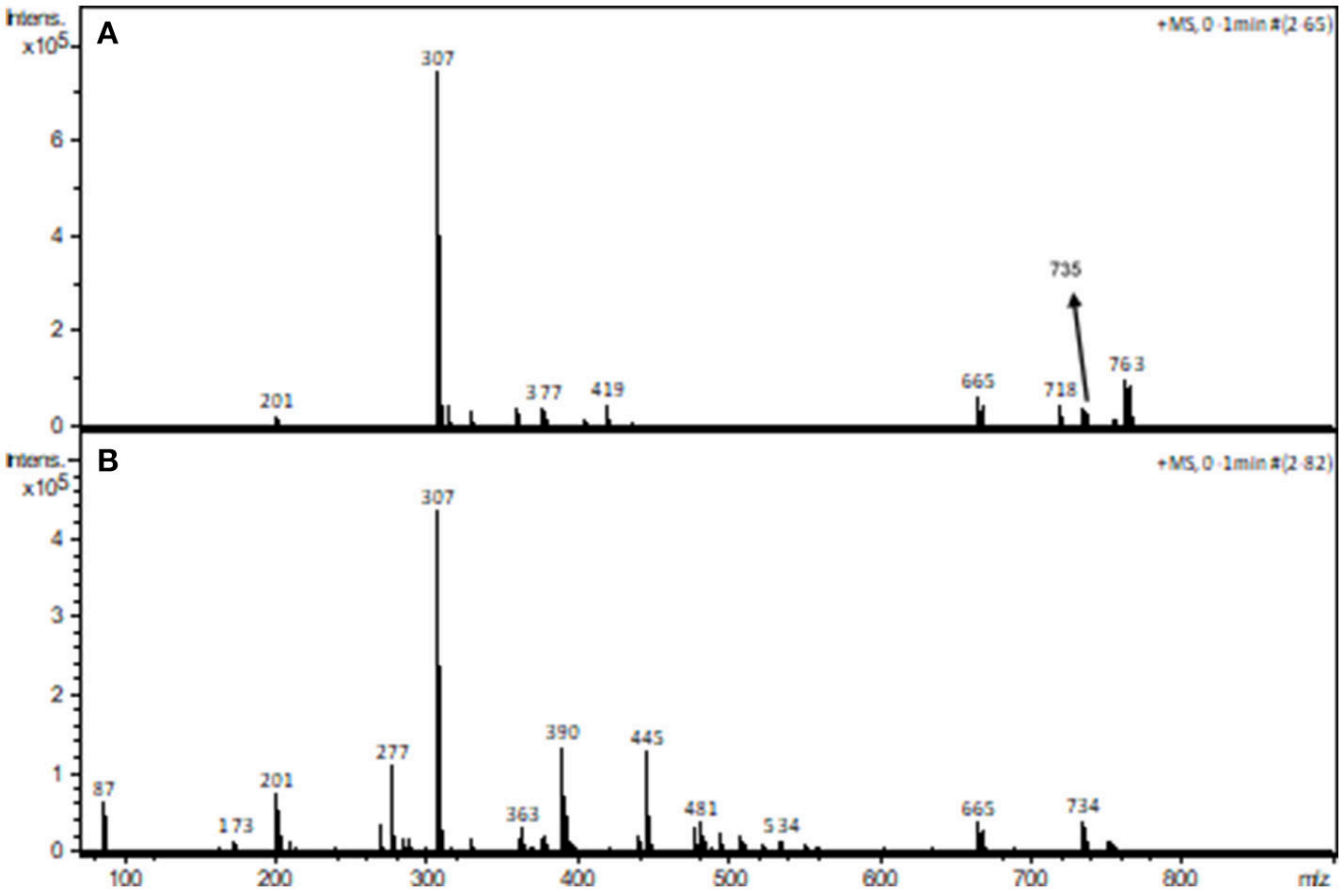
ESI-(+)-MS spectra of complex **1** in the absence **(A)** and in the presence **(B)** of piperazine in CH_3_CN/H_2_O.

The interaction between piperazine and complex **1** was also investigated using ESI-(+)-MS spectrometry. Figure 8 shows the spectra of the pure compound (A) and in the presence of piperazine (B).

**Figure 9:**
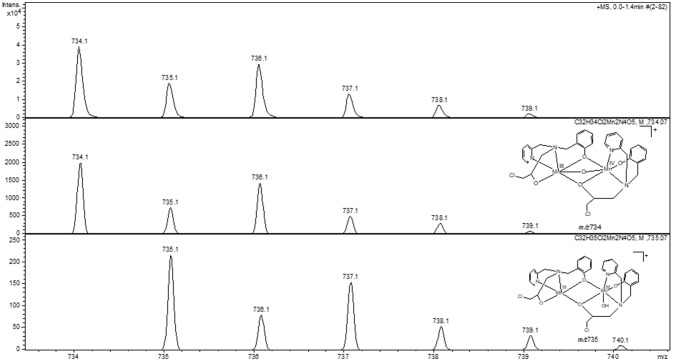
Experimental **(Top)** and calculated **(Middle, Bottom)** isotopic patterns for the ion at *m/z* 734. Proposed structures for the species are also shown.

The peak assignment of **1** was discussed above. In the presence of piperazine the base peak is still at *m/z* 307. However, some new species appear, including those at *m/z* 390, 445 (see Figures ESI15–16) and 734. Of particular relevance is the peak at *m/z* 734, which is absent in the spectrum of **1**. The calculated and experimental isotopic patterns for this peak are shown in Figure 9. The best simulation (position and intensity) was obtained by assuming the presence of two overlapping species: [(BPClNOL)Mn(III)-(μ-O)-Mn(IV)(BPClNOL)]^+^ with *m/z* 734 and [Mn_2_(III)(L)_2_(OH)]^+^ with *m/z* 735. The last signal was also observed in the mass spectrum of **1** (see above) while the signal at *m/z* 734 is a new species formed in the reaction between **1** and piperazine. Thus, in agreement with the UV-Vis spectral data (Figure 7), mass spectrometry supports a mechanism whereby piperazine induces the oxidation of **1** to a high-valent Mn(III)-(μ-O)-Mn(IV) species with *m/z* 734.

**Figure 10:**
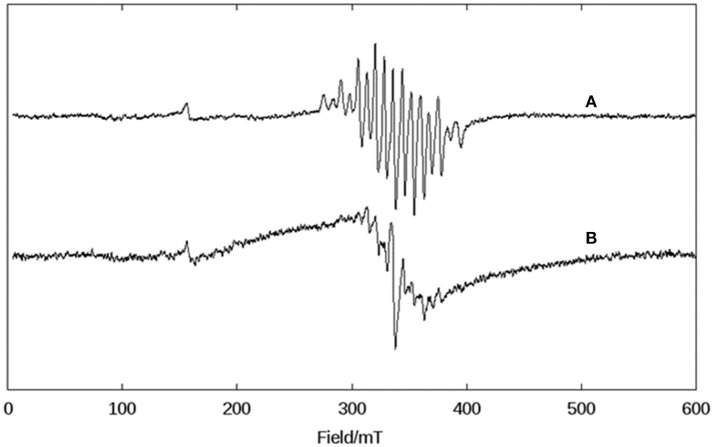
X-band CW EPR spectra of **1** in CH_3_CN at 140 K. **(A)** After the addition of piperazine and **(B)** after the addition of H_2_O_2_ to a solution containing the complex and piperazine.

The complex formed upon mixing **1**, piperazine and H_2_O_2_ was also probed by EPR spectroscopy. As discussed above, when **1** is exposed to H_2_O_2_ no oxygen production is observed. Not surprisingly, thus, the EPR spectrum of **1** in CH_3_CN and in the presence of H_2_O_2_ is virtually identical to that of the complex alone (i.e., Figure 3, bottom). However, when piperazine is added to **1**, an immediate change occurs that is consistent with the formation of a species containing a Mn(III)-(μ-O)-Mn(IV) center (Figure 10A); the relevant EPR spectrum has a 16-line feature that is typical of mixed-valent Mn(III)Mn(IV)-μ-oxo-bridged species (Horner et al., 1999; Dubois et al., 2008; Jiang et al., 2009; Mitić et al., 2009) supporting the data observed by UV-Vis and ESI-MS. The experimental spectrum could be simulated (see ESI, Figure ESI17) using the same equation and parameters employed in the simulation of the spectrum obtained for the reaction between **1** and superoxide (Figures 5, ESI12).

Upon the addition of H_2_O_2_ the spectrum of Mn(III)-(μ-O)-Mn(IV) changes with the loss of some resonances (Figure 10B). Now, at least 10 lines are observed, which suggests the formation of a new chemical species. We tentatively assign this new intermediate as a Mn(II)Mn(III) species, since this compound type has been described as presenting a 12 line spectrum (Larson et al., 1992; Gelasco et al., 1997; Mitić et al., 2009; Smith et al., 2009).

Previously, we have reported an investigation of the CAT activity of complex **2** (Figure 1). Similar to complex **1**, a Mn(III)-Mn(IV) intermediate was observed by EPR (Lessa et al., 2009). In the present study the Mn(III)Mn(IV) compound is formed after the interaction of a mixed-valent Mn(II)Mn(III) species with piperazine under aerobic conditions. In contrast, for **2** the formation of a Mn(III)Mn(IV) species was shown to be due to the reaction between a homo-valent Mn(II) complex and H_2_O_2_. Another difference is that for **1**, only one μ-oxo bridge is proposed to be present, while for **2**, two μ-oxo bridges connect the metal ions.

It has been shown that the presence of a base (e.g., imidazole, trimethylamine) increases the CAT activity of synthetic compounds and it has been proposed that this increase is associated with the deprotonation of the H_2_O_2_ molecule (Devereux et al., 2002; Grau et al., 2014; Kose et al., 2015). While piperazine is a base it is also a chelator, and hence it could promote CAT reactivity using either property. However, our combined data strongly support the interpretation that piperazine induces changes in the oxidation state and in the coordination environment of the manganese centers in complex **1**. A plausible pathway is by promoting the deprotonation of the alcohol function, which changes the Lewis acidity of the Mn(II), leading to its oxidation in the presence of O_2_ to generate the Mn(III)-oxo-Mn(IV) species. In order to substantiate this hypothesis, we performed a test reaction between **1** and H_2_O_2_, but using triethylamine instead of piperazine (see ESI Figure ESI18). The result revealed a similar catalase activity, suggesting that both piperazine and triethylamine act as a base. EPR studies of the interaction between **1** and triethylamine revelead the formation of a Mn(III)-oxo-Mn(IV) unit, since a 16-line EPR spectrum was observed (Figure ESI19), confirming that both bases (piperazine, triethylamine) induce the formation of the same intermediate.

### Mechanistic proposals

The evaluation of the IC_50_ has shown that **1** is able to prevent NBT reduction in the presence of superoxide anions (Table 3). Furthermore, we have demonstrated that **1** interacts directly with this ROS in DMSO, promoting its decomposition, as seen by the disappearance of the superoxide radical signal (Figure 5). As a consequence of the reaction, the characteristic 6-line EPR spectrum from a Mn(II) complex was transformed into a 16-line one, which is typical of a Mn(III)Mn(IV)-coupled species containing a μ-oxo bridge (Larson et al., 1992; Gelasco et al., 1997; Lessa et al., 2009; Mitić et al., 2009; Smith et al., 2009). Additionally, the EPR spectra of **1** in water and in DMSO are equivalent, indicating that the dinuclear structure is broken in solution, generating Mn(II) and Mn(III) species. Therefore, it is plausible to assume that the chemical species present in the water solution employed in the catalytic study is similar to the one present in the DMSO solution employed for the superoxide dismutation study monitored via EPR. Thus, we propose that in the initial step of the reaction the superoxide anion reacts with two mononuclear Mn(II) species (observed by EPR), resulting in a peroxo complex (Equation 1) containing a Mn(II)Mn(III) center, which subsequently is further oxidized to a high-valent Mn(III)Mn(IV) species (Equation 2). The two electrons involved in this process are necessary to reduce the peroxide species to water, leading to the formation of a μ-oxo bridge which was detected by EPR (Equation 2). These two steps are similar to those proposed for the compounds [Mn(BIG)(H_2_O)_2_]^+^ and [Mn(IPG)(MeOH)] (Policar et al., 2001). The reaction of this high-valent species with another superoxide generates molecular oxygen with the concomitant formation of a Mn(III)-oxo-Mn(III) species (Equation 3). This homo-valent species may be re-oxidized by the reaction with another superoxide molecule, forming H_2_O_2_ and the high-valent Mn(III)Mn(IV) species in the process (Equation 4), which then enters the reaction again at the step described by Equation (3).

(1) $$\begin{array}{r} 2\text{Mn}\left(\text{II}\right)+{\text{O}}_{2}^{-}\text{➜Mn}\left(\text{II}\right)\text{Mn}\left(\text{III}\right)-\text{peroxide} \end{array}$$

(2) $$\begin{array}{r} \text{Mn}\left(\text{II}\right)\text{Mn}\left(\text{III}\right)-\text{peroxide➜Mn}\left(\text{III}\right)-\text{oxo}-\text{Mn}\left(\text{IV}\right)+{\text{H}}_{2}\text{O} \end{array}$$

(3) $$\begin{array}{r} \text{Mn}\left(\text{III}\right)-\text{oxo}-\text{Mn}\left(\text{IV}\right)+{\text{O}}_{2}^{-}\text{➜Mn}\left(\text{III}\right)-\text{oxo}-\text{Mn}\left(\text{III}\right)+{\text{O}}_{2} \end{array}$$

(4) $$\begin{array}{l} \text{Mn}\left(\text{III}\right)-\text{oxo}-\text{Mn}\left(\text{III}\right)+{{\text{O}}_{2}}^{-}\text{➜Mn}\left(\text{III}\right)-\text{oxo}-\text{Mn}\left(\text{IV}\right)+\text{peroxide} \end{array}$$

Commonly SOD mimetics are already in the active form to promote the reduction of superoxide to peroxide and molecular oxygen. For example, Mn-porphyrins and Mn-salen compounds show SOD like activity in which the oxidation state changes between III/II (Shin et al., 2010; Signorella et al., 2018). For such systems the catalytic process usually involves a step for the oxidation of superoxide, thus forming molecular oxygen, and another step for the reduction of superoxide, resulting in the formation of hydrogen peroxide (ping-pong mechanism). On the other hand, complexes containing tripodal amine ligands sometimes need to be activated to promote the disproportionation of ROS (Lessa et al., 2009; Signorella et al., 2018) as observed for the compounds [Mn(BIG)(H_2_O)_2_]^+^ and [Mn(IPG)(MeOH)] (Policar et al., 2001), which, after reacting with superoxide, are transformed into the dinuclear species Mn^III^-(μ-O)_2_-Mn^IV^. The same behavior was described for the mononuclear compounds [Mn^II^(N4py)(OTf)](OTf) (Leto et al., 2013).

**Figure 11:**
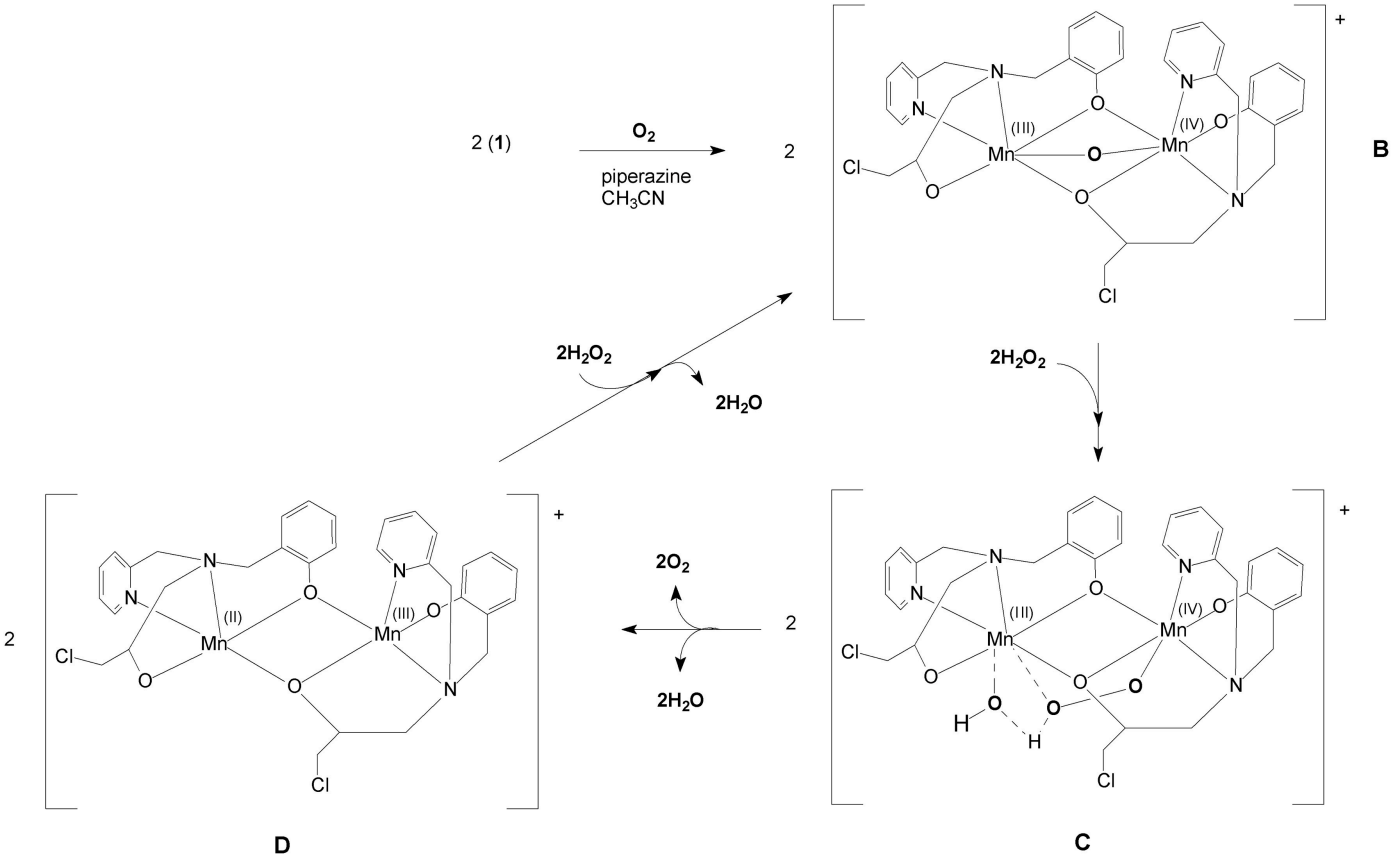
Mechanistic proposal for the H_2_O_2_ disproportionation reaction promoted by complex **1** based on EPR, ESI-(+)-MS and kinetic data.

A proposed model for the CAT mechanism employed by **1** is shown in Figure 11. When **1** is placed in contact with piperazine, an oxidation process occurs, transforming the system to Mn(III)-(μ-O)-Mn(IV). Thus, we propose that **1** is transformed to B, the mixed-valent μ-oxo bridged species that was observed by UV-Vis, ESI-MS and EPR. The next step (B➜C in Figure 11) occurs after the addition of H_2_O_2_. The formation of a new Mn(III)Mn(IV) complex is proposed, in which a hydroperoxide molecule displaces the μ-oxo group. This interpretation would explain why the 16-line EPR feature disappears in the presence of H_2_O_2_ (Figure 10). In this unstable arrangement, the peroxide molecule may transfer two electrons to the binuclear Mn(III)Mn(IV) cluster, resulting in the release of molecular oxygen and water and the generation of a Mn(II)Mn(III) center (D in Figure 11). Further reaction with H_2_O_2_ results in the formation of the Mn(III)-oxo-Mn(IV) species again (B in Figure 11) and release of H_2_O.

The proposed mechanism for the CAT activity presented by **1** is significantly different from that proposed for **2. 1** needs to be transformed into the active species by piperazine, forming a Mn(III)-(μ-O)-Mn(IV). In contrast, **2** reacts directly with H_2_O_2_ and a Mn(III)-(μ-O)_2_-Mn(IV) intermediate is formed.

## Conclusions

In this study we have reported the synthesis and characterization of an unusual mixed-valent manganese compound which forms a polymeric linear chain in the solid state. It consists of Mn(II)Mn(III) subunits, in which the manganese ions are connected by a phenoxide and an alkoxide bridge (Figures 1, 2). The subunits are linked via chloro bridges. In the solid state, **1** shows two distinct magnetic interactions. An antiferromagnetic one [*J* = −5.224(13) cm^−1^] is observed between the monomers (through chloro bridges) and a ferromagnetic coupling [*J* = +0.076(13) cm^−1^] is observed in the monomeric unit (via the alkoxide/phenoxide bridges). The latter interaction supports the attribution that the signal observed in the EPR spectrum at g around 7 is a result of a ferromagnetic coupled Mn(III)Mn(II) system, and therefore, that the compound remains as a dinuclear center in CH_3_CN. In contrast, EPR spectroscopy has revealed that in DMSO and H_2_O solutions the dinuclear structure is broken, leading to monomeric Mn(II) and Mn(III) units (Figure 3). Importantly, in aqueous environment, the compound has dual antioxidant activity, i.e., it acts both as a catalase and as superoxide dismutase. For the reaction with superoxide, a Mn(III)-(μ-O)-Mn(IV) species was identified as intermediate by EPR. With respect to the catalase activity, we found that the resting Mn(II)Mn(III) species is active only in presence of a base such as piperazine or trimethylamine. It was shown that piperazine promotes the formation of an active Mn(III)-(μ-O)-Mn(IV). An intermediate of the reaction of this Mn(III)-(μ-O)-Mn(IV) species with H_2_O_2_ could also be detected by EPR, suggesting that the formation of a Mn(II)Mn(III) species that promote the CAT activity of **1**, involves a Mn^II^Mn^III^/Mn^III^Mn^IV^ redox couple.

## Author's note

Catalase activity evaluated by UV-Vis, the comparison of the catalase activity in the presence of piperazine and triethylamine, ESI-(+)-MS and EPR data are presented as supporting information. Crystallographic data for the structure reported in this paper have been deposited with the Cambridge Crystallographic Data Centre as supplementary publication. Deposition number: 1478947. Copies of the data can be obtained free of charge from the CCDC at www.ccdc.cam.ac.uk.conts/retrieving.html/ or from the Cambridge Crystallographic Data Centre (CCDC), 12 Union Road, Cambridge CB2 IEZ, UK; fax: 44(0) 1223-336033; e-mail: deposit@ccdc.cam.ac.uk.

## Author contributions

RC, SF, and CP carried out the syntheses, the characterization of the compounds and the kinetics experiments. JH, CN, and RF carried out the EPR experiments, and the simulations of the data. JR performed the x-ray experiments and the data treatment. PC and AR were responsible for the magnetism measurements and data interpretation. GS contributed in the analyses and discussion of kinetic data. CF and AH conceived and planned the experiments, supervised the progress of this work, and took the lead in writing the manuscript. All authors discussed the results and contributed to the final manuscript.

### Conflict of interest statement

The authors declare that the research was conducted in the absence of any commercial or financial relationships that could be construed as a potential conflict of interest.

## Footnotes

^1^https://www.dojindo.com/Shared/Protocol/SpinTrapApplication.pdf Retrieved: 09/11/2017.
