# Supplementary material for: A New Mixed-Valence Mn(II)Mn(III) Compound With Catalase and Superoxide Dismutase Activities
Source: Front Chem. 2018 Nov 5;6:491. doi: 10.3389/fchem.2018.00491 (PMC6231112; doi:10.3389/fchem.2018.00491)
Supplement: Supplementary file 1 [file Data_Sheet_1.PDF]

Electronic Supplementary Information

A new mixed-valence Mn(II)Mn(III) compound with catalase and superoxide dismutase activities

Rafael O. Costa,[a] Sarah S. Ferreira,[b] Crystiane A. Pereira,[a] Jeffrey R. Harmer,[c] Christopher J. Noble,[c] Gerhard Schenk,[d] Roberto W. de A. Franco,[e] Jackson A. L. C. Resende,[f] Peter Comba,[g] Asha E. Roberts,[g] Christiane Fernandes,[a, *] Adolfo Horn Jr,[a, *]

[a] Laboratório de Ciências Químicas, Universidade Estadual do Norte Fluminense, 28013-602, Campos dos Goytacazes/RJ, Brazil

[b] Instituto Federal Fluminense, Campus Centro, 28013-602, Campos dos Goytacazes/RJ, Brazil

[c] Centre for Advanced Imaging, The University of Queensland, Brisbane, QLD 4072, Australia

[d] School of Chemistry and Molecular Biosciences, The University of Queensland, Brisbane, QLD 4072, Australia

[e] Laboratório de Ciências Físicas, Universidade Estadual do Norte Fluminense, 28013-602, Campos dos Goytacazes/RJ, Brazil

[f] Laboratório de Difração de Raios X, Universidade Federal Fluminense, 24020-150, Niterói/RJ, Brazil

[g] Anorganisch-Chemisches Institut, Universität Heidelberg, INF 270, D-69120 Heidelberg, Germany and Interdisziplinäres Zentrum für Wissenschaftliches Rechnen (IWR), D-69120 Heidelberg, Germany

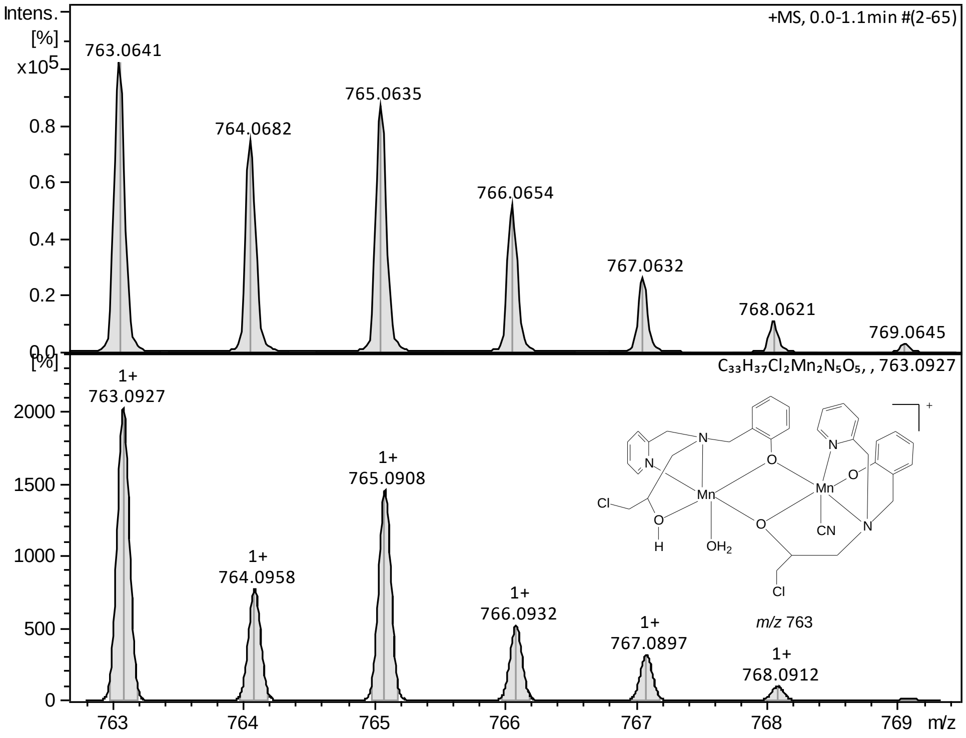

Figure ESI1. Experimental (top) and calculated (bottom) isotopic pattern for the ion with m/z 763. A proposal for the structure is presented.

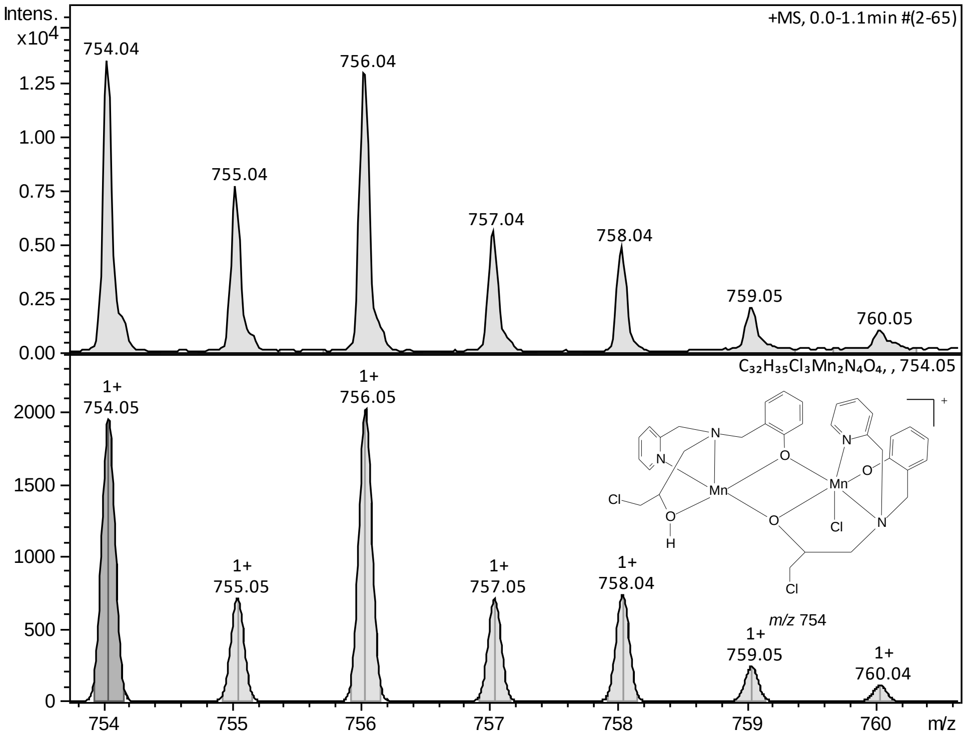

Figure ESI 2. Experimental (top) and calculated (bottom) isotopic pattern for the ion with m/z 754. A proposal for the structure is presented.

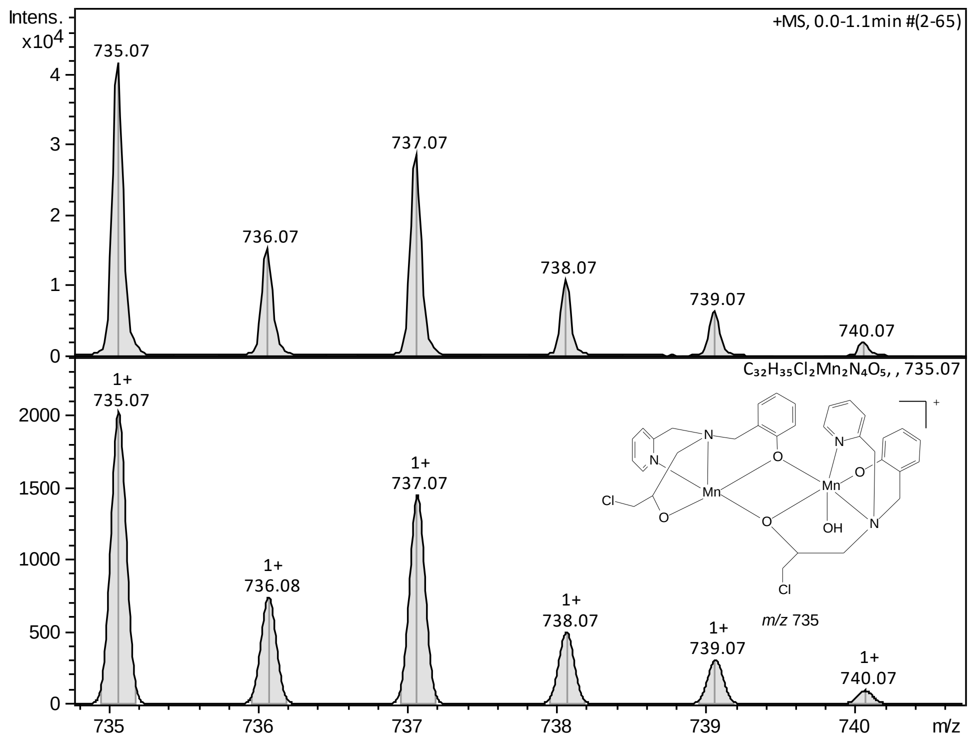

Figure ESI 3. Experimental (top) and calculated (bottom) isotopic pattern for the ion with m/z 735. A proposal for the structure is presented.

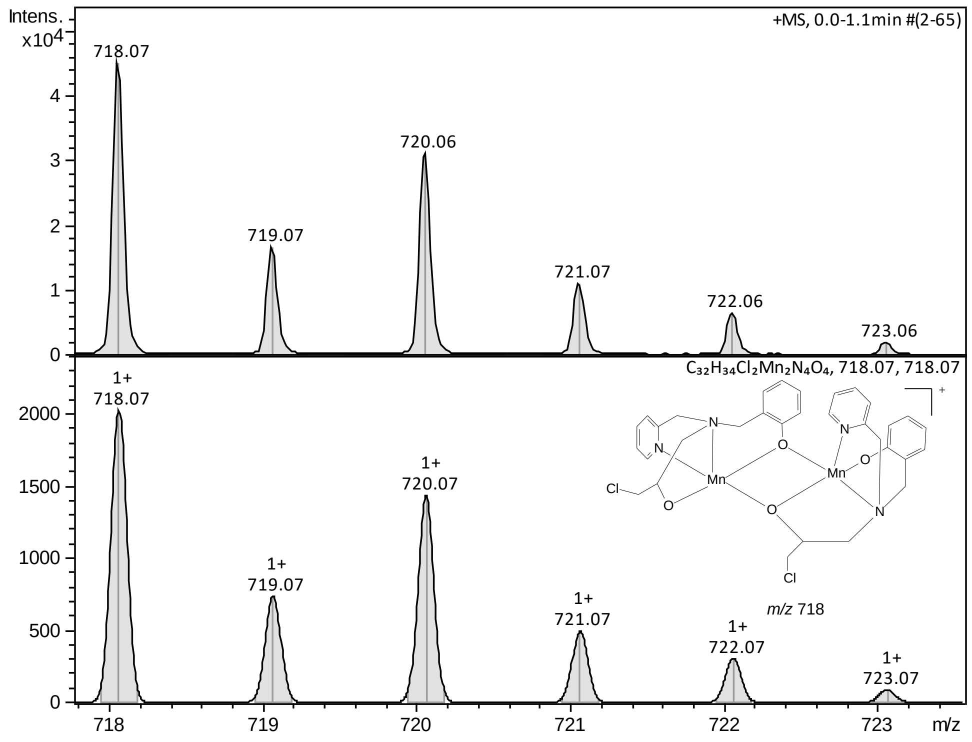

Figure ESI 4. Experimental (top) and calculated (bottom) isotopic pattern for the ion with m/z 718. A proposal for the structure is presented.

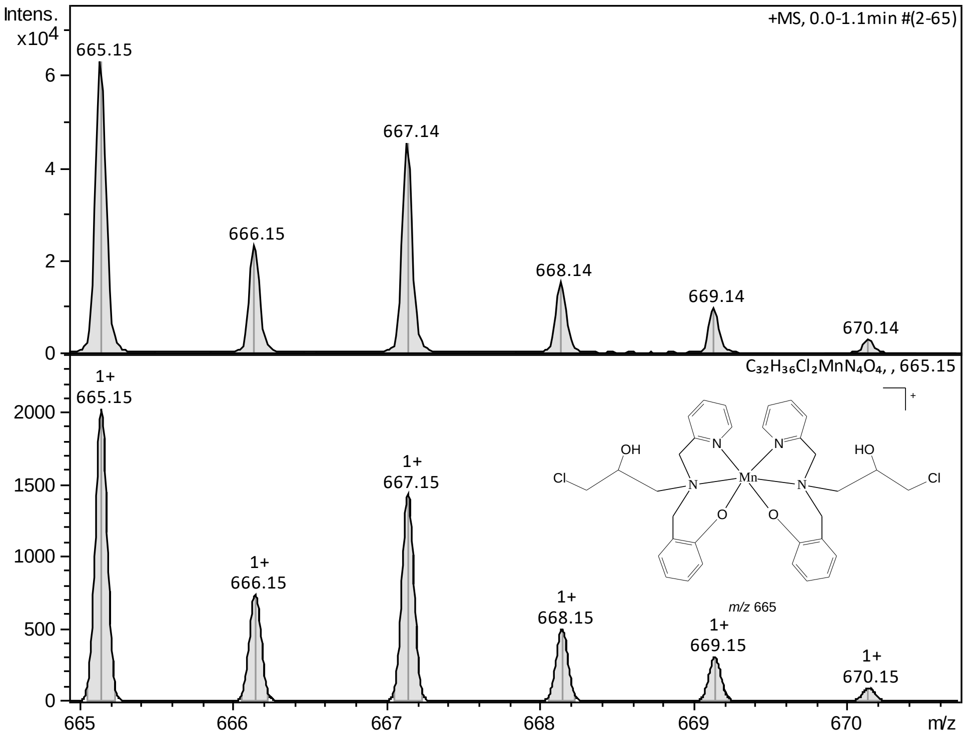

Figure ESI 5. Experimental (top) and calculated (bottom) isotopic pattern for the ion with m/z 665. A proposal for the structure is presented.

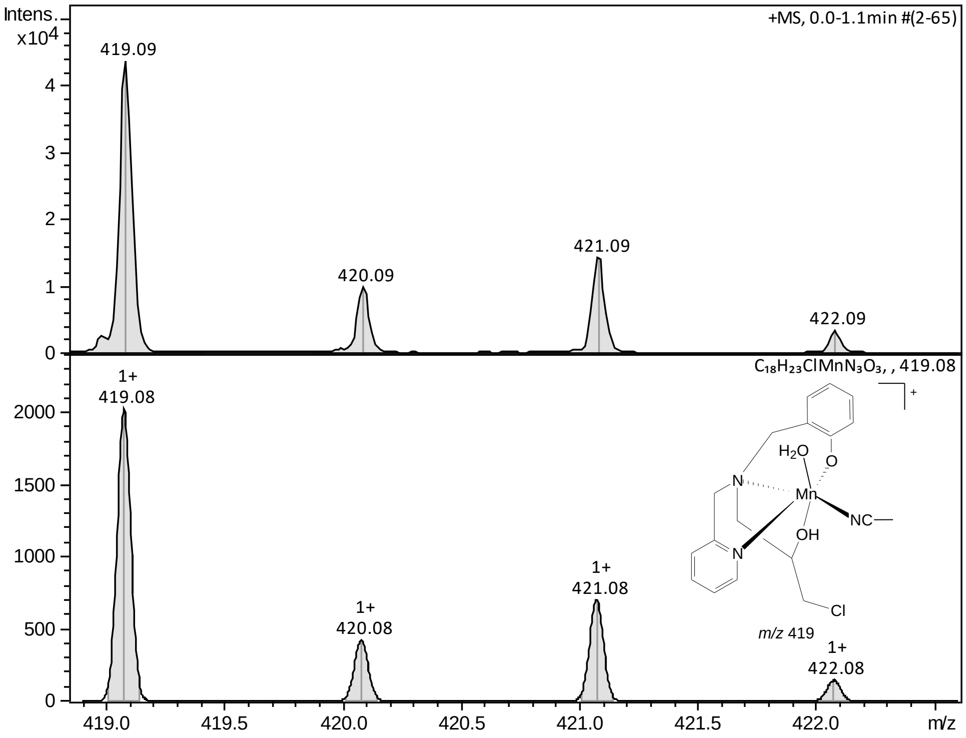

Figure ESI 6. Experimental (top) and calculated (bottom) isotopic pattern for the ion with m/z 419. A proposal for the structure is presented.

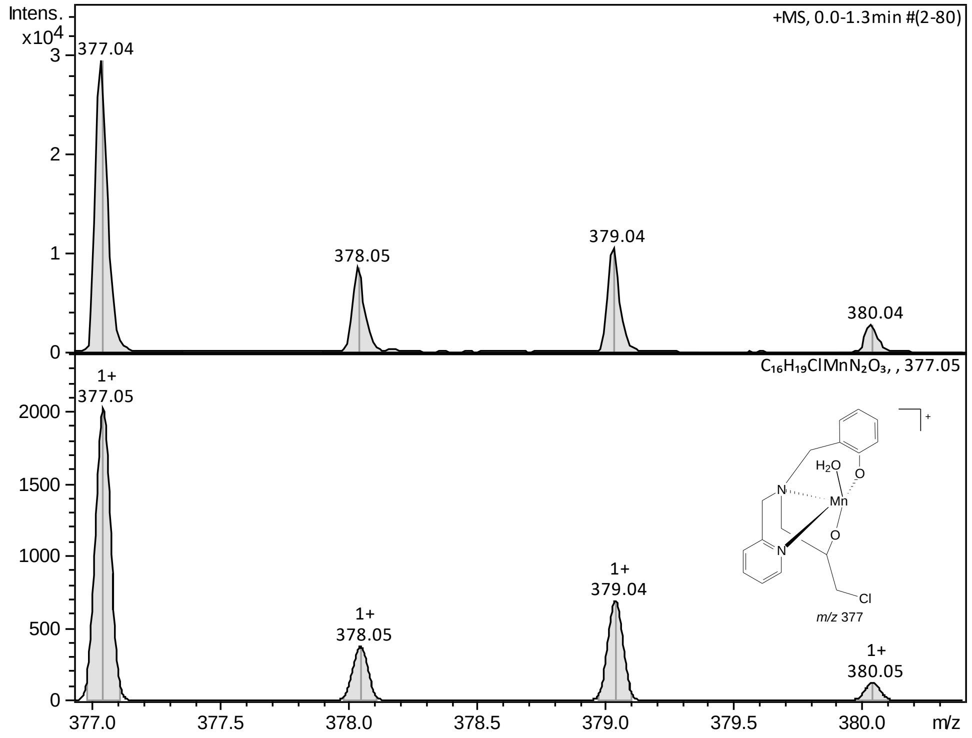

Figure ESI 7. Experimental (top) and calculated (bottom) isotopic pattern for the ion with m/z 377. A proposal for the structure is presented.

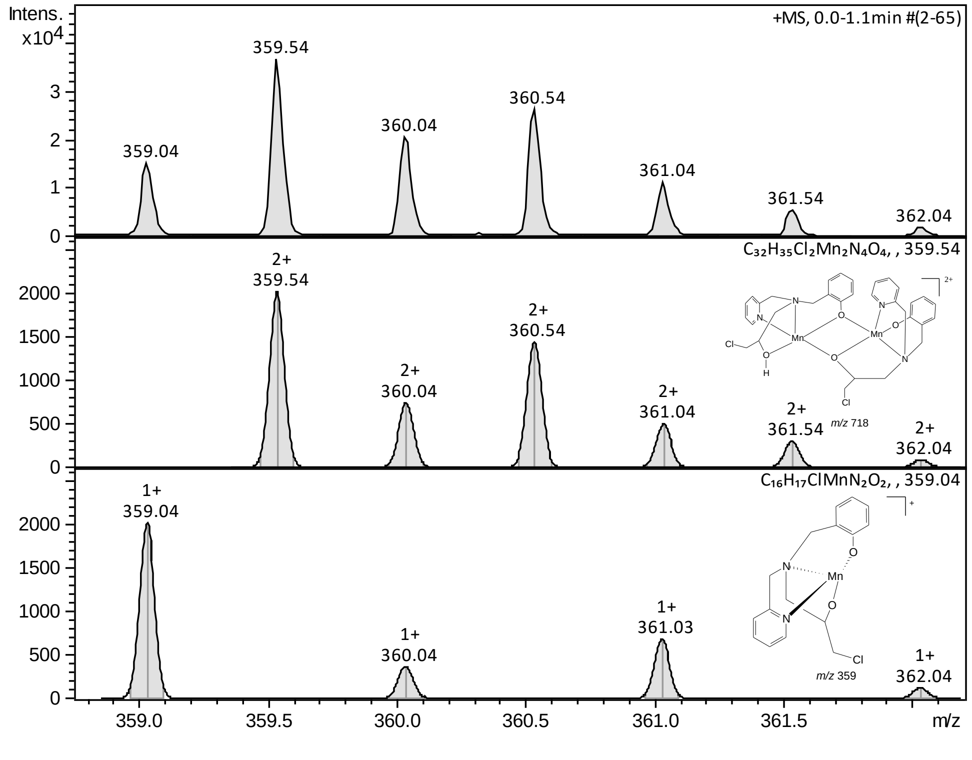

Figure ESI 8. Experimental (top) and calculated (bottom) isotopic pattern for the ion with m/z 359, which agrees with the overload of two species. A proposal for the structures is presented.

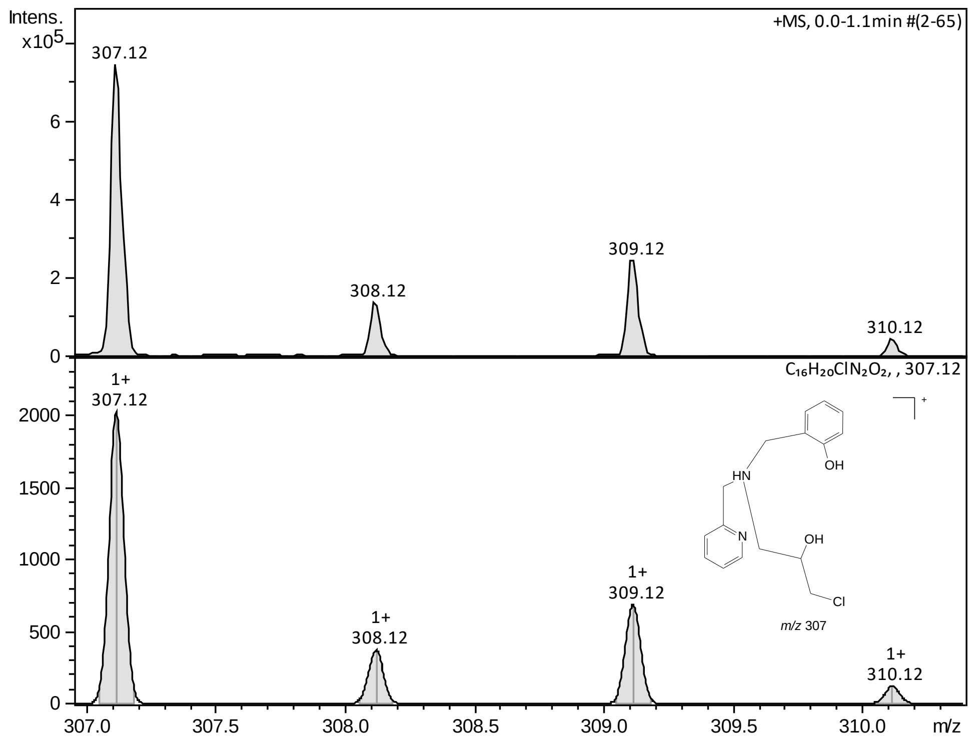

Figure ESI 9. Experimental (top) and calculated (bottom) isotopic pattern for the ion with m/z 307. A proposal for the structures is presented.

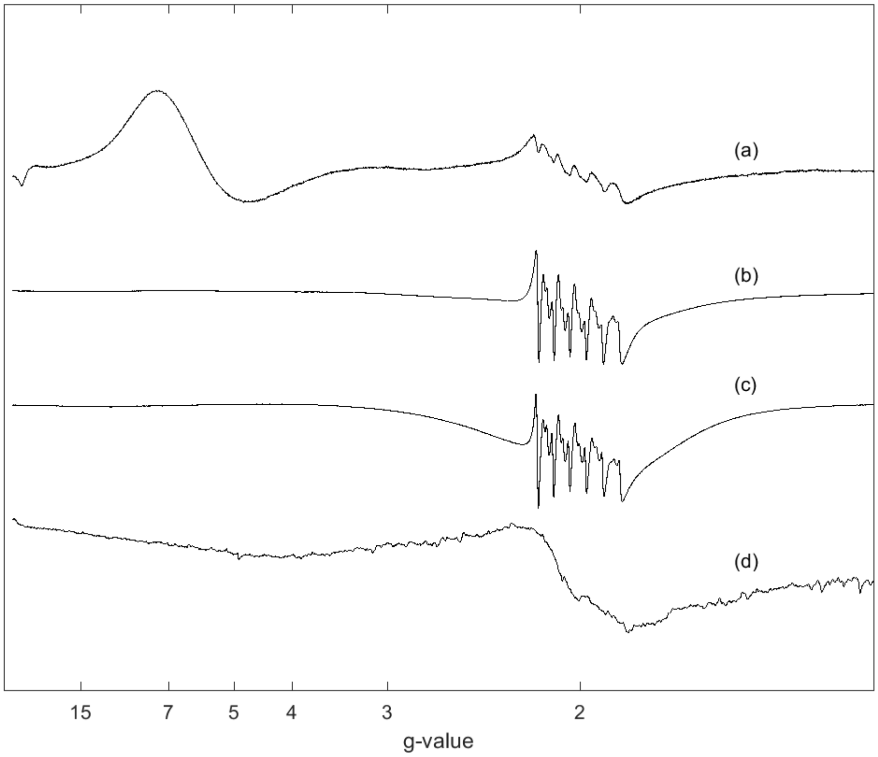

Figure ESI 10. EPR spectra of **1** in frozen solution at 1.8 K of a) CH_3CN, b) DMSO, c) H_2O, d) solid state.

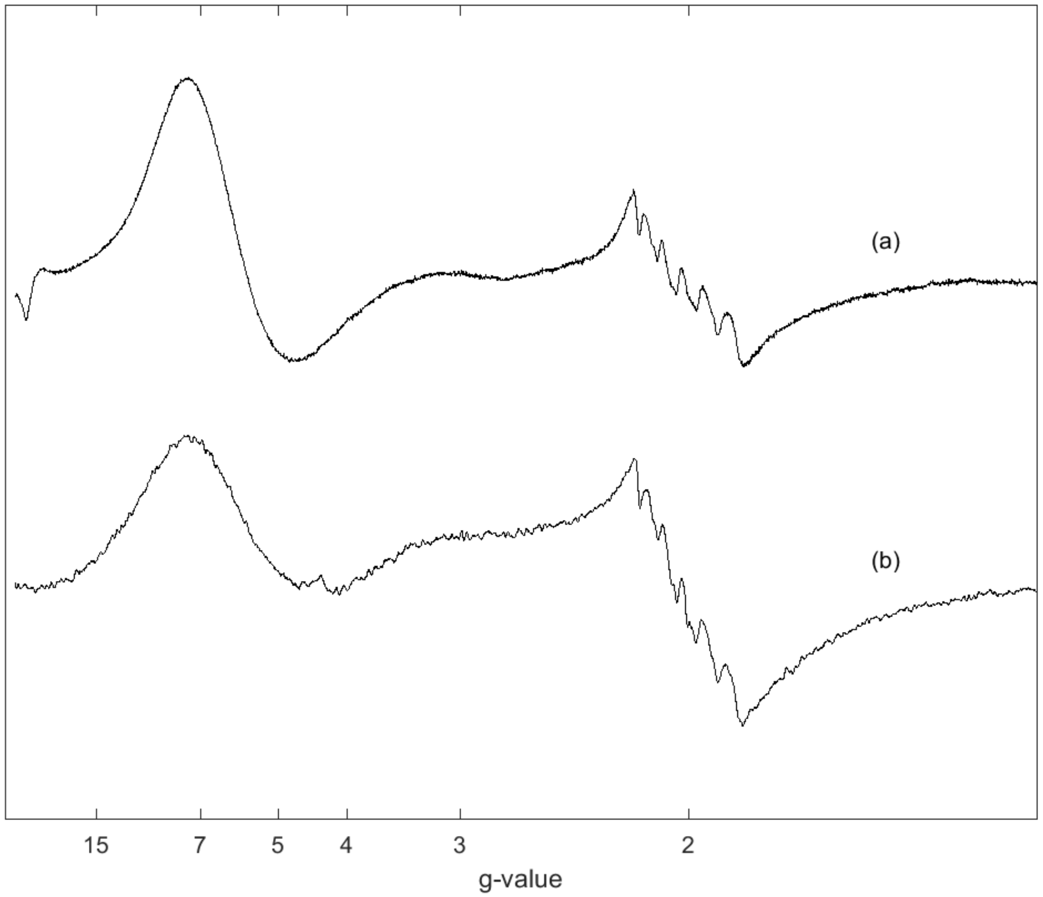

Figure ESI 11. EPR spectra of **1** in CH_3CN at (a) 1.8K and (b) 140K.

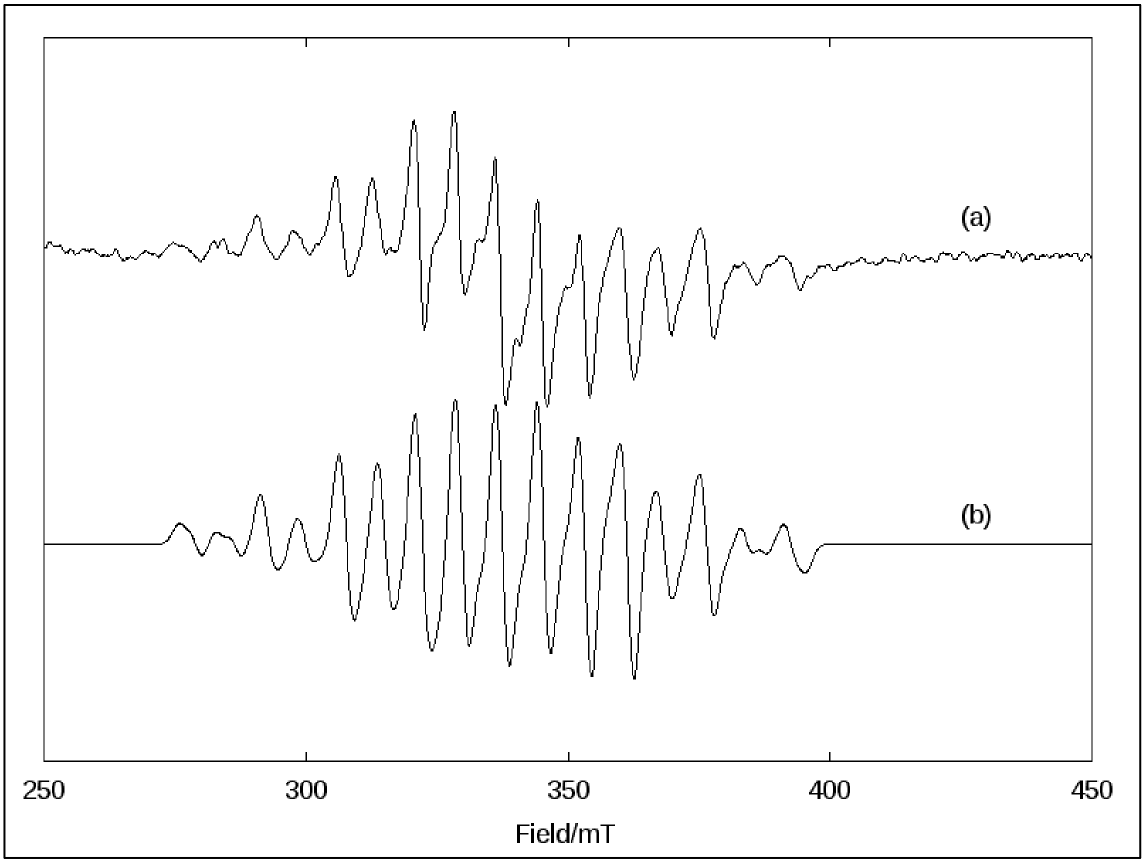

Figure ESI 12.(a) Experimental and (b) simulated EPR spectra of complex **1** immediately after the interaction with superoxide in DMSO at 140 K.

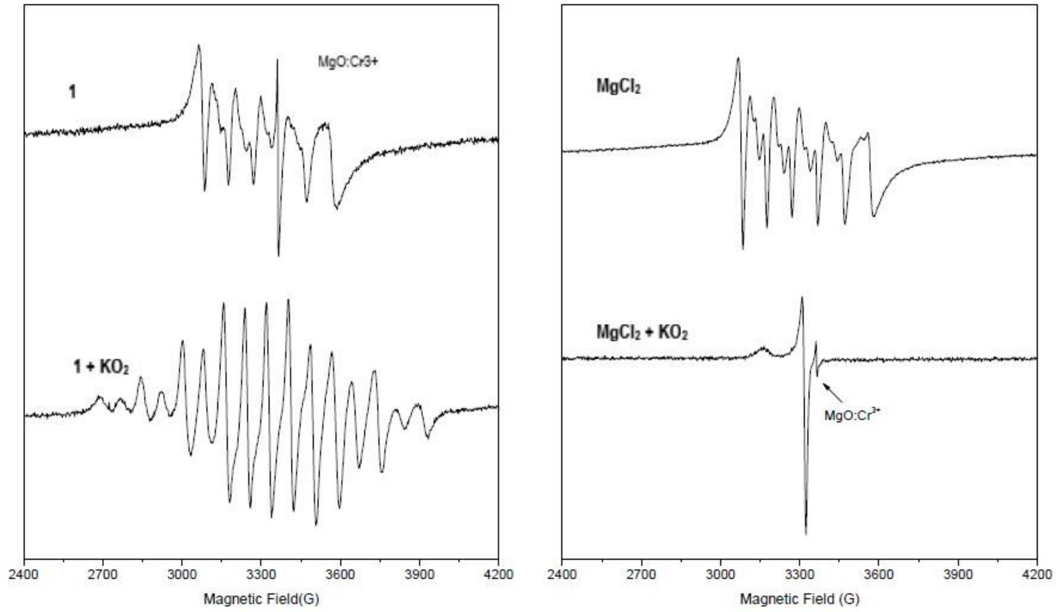

Figure ESI 13. EPR spectra in DMSO of compound **1** before (left, top) and after reaction with KO_2 (left, bottom). EPR spectra of $MnCl_2.4H_2O$ before (right, top) and after (right, bottom) reaction with KO_2.

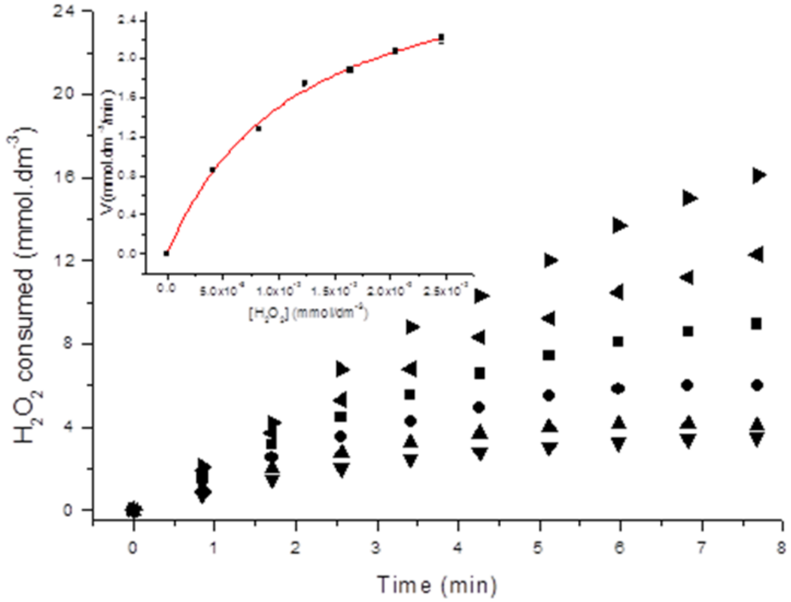

Figure ESI 14. Rates of H_2O_2 consumption at [**1**] = $2.1x10^{-5}$ mol dm^{-3} and different concentration of H_2O_2 ($2.46x10^{-2}$, ► ; $2.05x10^{-2}$, ◄ ; $1.64x10^{-2}$, ■ ; $1.23x10^{-2}$, ● ; $8.2x10^{-3}$, ▲; $4.1x10^{-3}$ M, ▼). The inset shows the dependence of the rate on substrate concentration, together with a fit to the Michaelis–Menten equation.

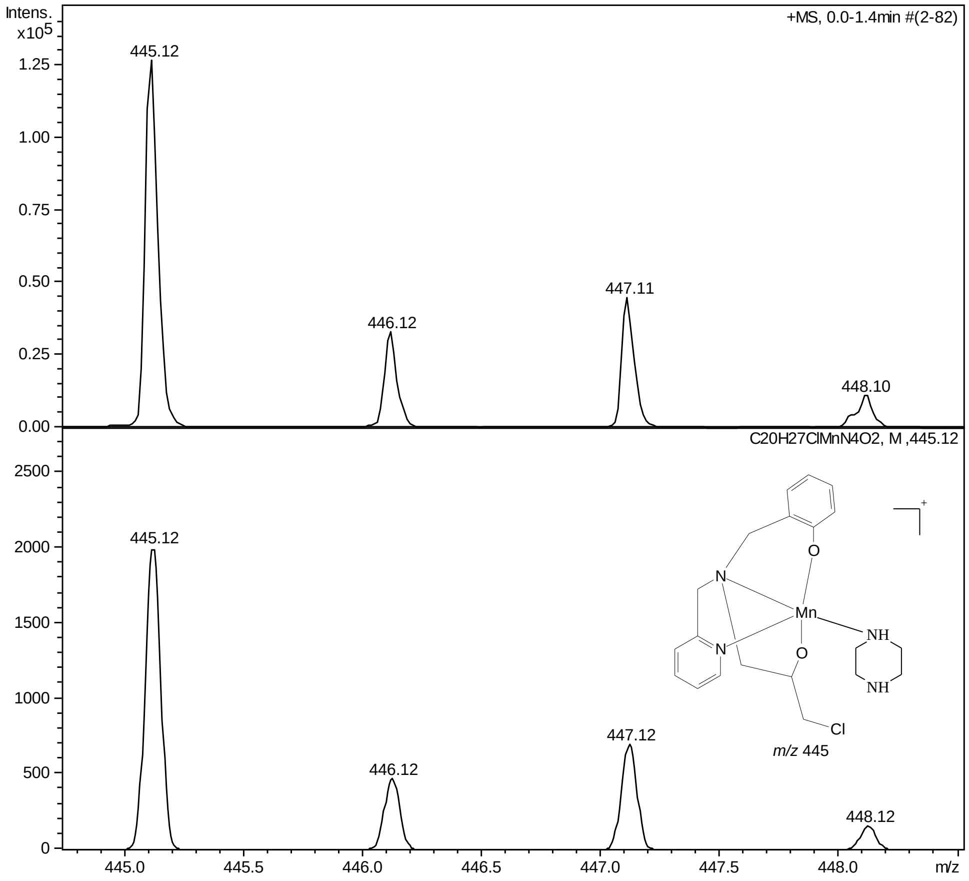

Figure ESI 15. Experimental (top) and calculated (bottom) isotopic pattern for the ion with m/z 445 observed in the reaction between **1** and piperazine. A proposal for the structure is presented.

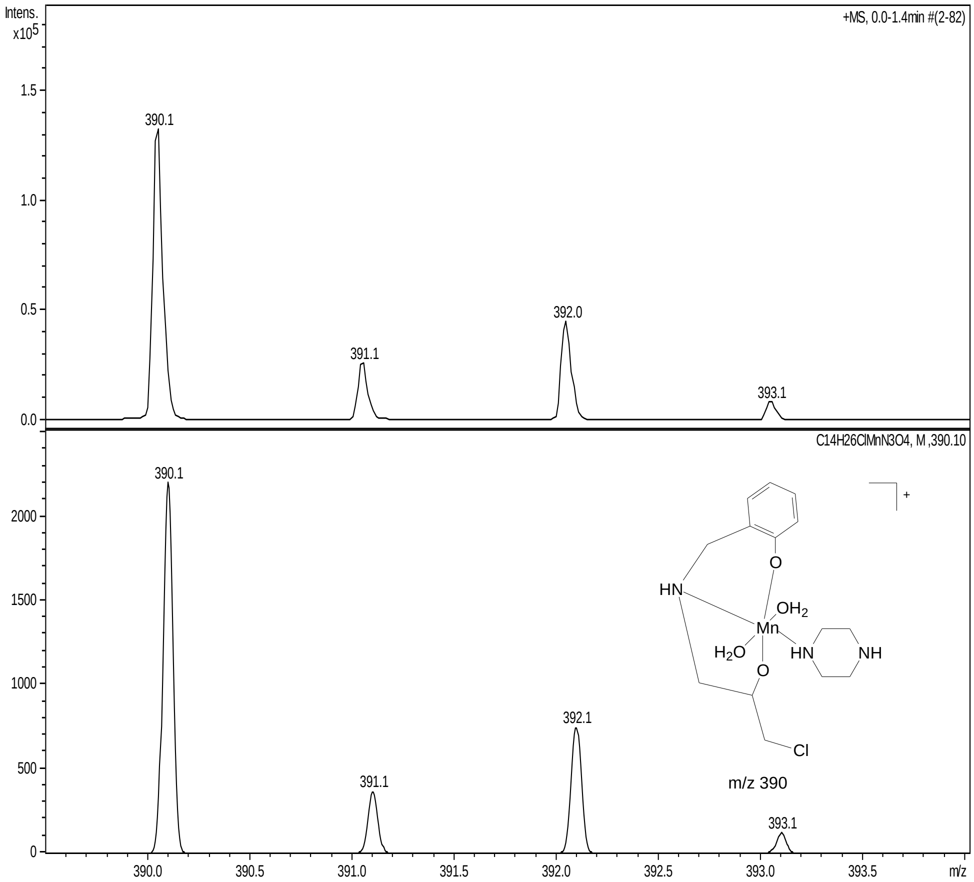

Figure ESI 16. Experimental (top) and calculated (bottom) isotopic pattern for the ion with m/z 390 observed in the reaction between **1** and piperazine. A proposal for the structures is presented.

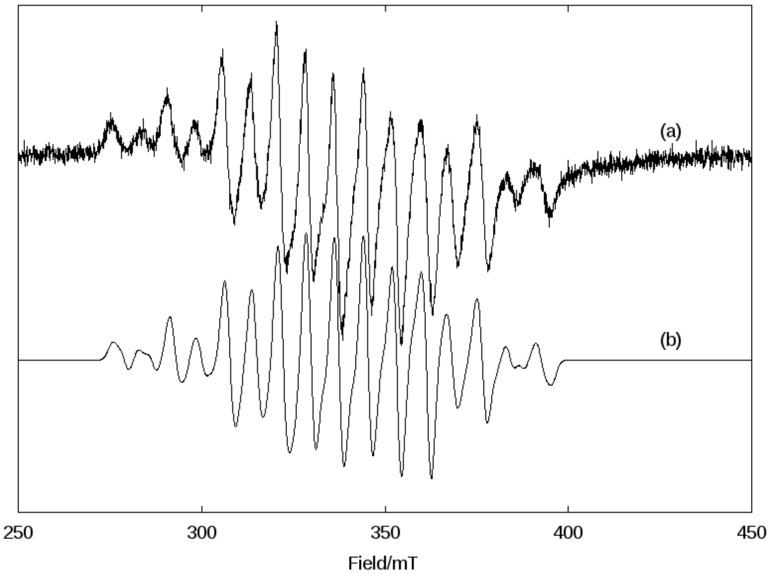

Figure ESI 17. Experimental EPR spectrum at 140 K (a) and corresponding simulation (b) of a Mn(III)-(μ-O)-Mn(IV) dimer formed upon the interaction of piperazine with **1**.

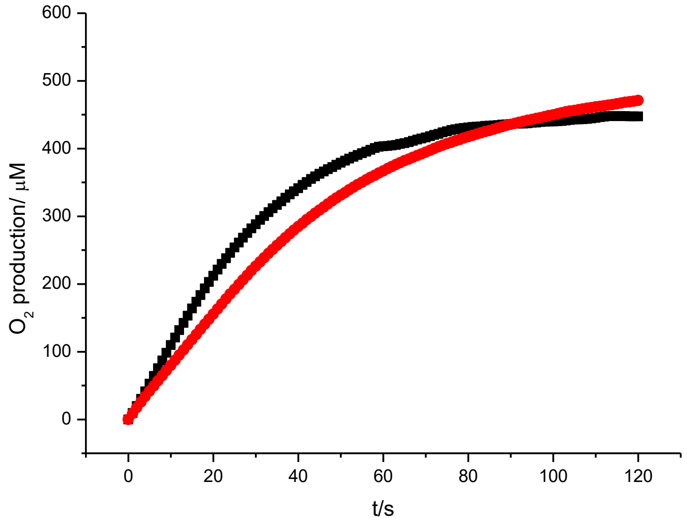

Figure ESI 18. Plot of the O_2 production promoted by **1** (22.7 μmol dm^{-3}), in the presence of piperazine (red) and trimethylamine (black) (4.5 mmol dm^{-3}) at $[H_2O_2]$ = 7.3 mM.

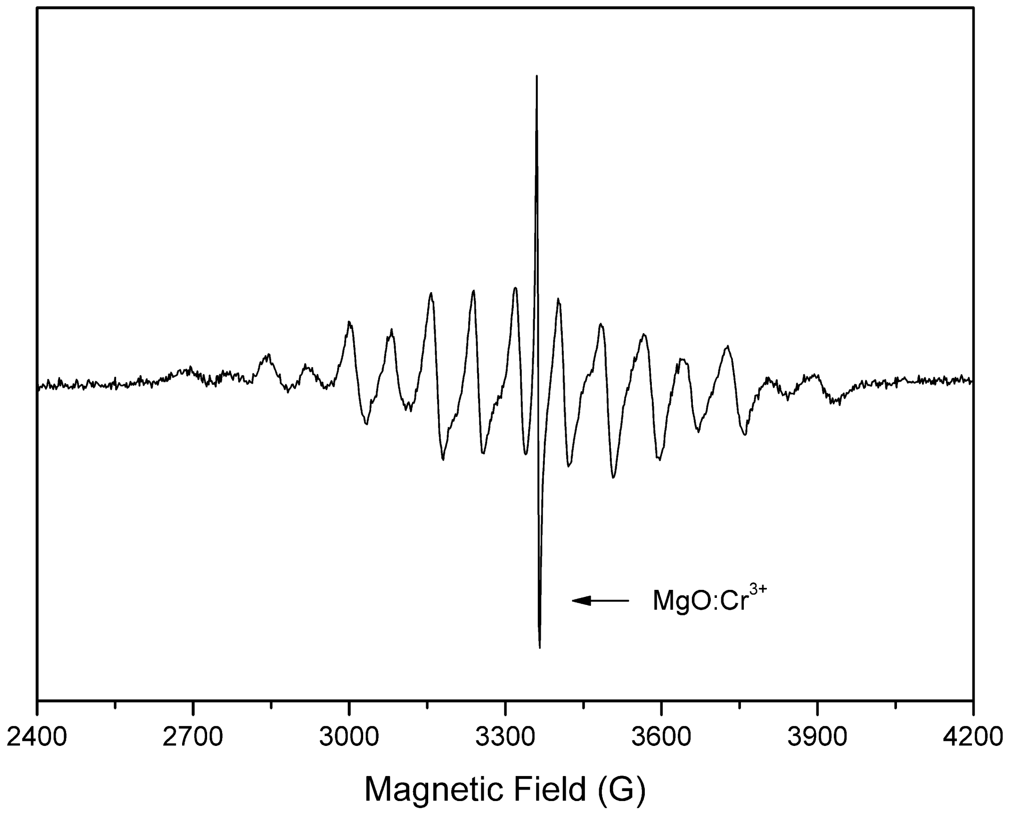

Figure ESI 19. EPR spectrum in CH_3CN at 110 K showing the formation of a Mn(III)-(μ-O)-Mn(IV) dimer upon the interaction of triethylamine with **1**.
